# A Physiological and Pathological Inquiry Concerning the Physical Characteristics of the Human Teeth and Gums, the Salivary Calculus, the Lips and Tongue, and the Fluids of the Mouth; Together with Their Respective Local and Constitutional Indications

**Published:** 1841-09

**Authors:** Chapin A. Harris

**Affiliations:** Professor of Practical Dentistry in the Baltimore College of Dental Surgery.


### ARTICLE III.

*A Physiological and Pathological Inquiry concerning the Physical Characteristics of the Human Teeth and Gums, the Salivary Calculus, the Lips and Tongue, and the Fluids of the Mouth; together with their respective local and constitutional indications.* Read before the American Society of Dental Surgeons, at their second annual meeting, held in Philadelphia, August 11, 1841. By CHAPIN A. HARRIS, M. D., D. D. S. Professor of Practical Dentistry in the Baltimore College of Dental Surgery.

*Mr. President and Gentlemen:—*

WILLING to contribute to the extent of my humble abilities, to promote the object of your association, I accepted the appointment with which you honored me at your first meeting, to prepare a "dissertation on some subject connected with dental theory or practice;" but the little leisure afforded from arduous professional and other duties, has prevented me from accomplishing the task, in as full and perfect a manner as I could have wished. Written in haste, without time for revision or correction, the paper which I am about to submit to your consideration, is, I am aware, far from faultless. The subjects embraced in it, though, perhaps, somewhat novel in their character, are, nevertheless, important; and if the imperfect manner in which they are here exhibited, meets your approval, I shall not regret having complied with your wishes.

### INTRODUCTION.

The means for the prevention and cure of disease, have, perhaps, been studied with more persevering and laborious industry, than any other branch of knowledge that has ever engaged the attention of the human mind. They have been sought by the learned and wise of almost every age and nation. With a view to their attainment, the vegetable and mineral kingdoms have each, in turn, been explored. Almost every flower, leaf and root of the former, and metal and earth of the latter, have been carefully analyzed, and the medicinal virtues of the components of each, anxiously tested. Nor has the pursuit stopped here. Anatomical and physiological investigations have been called into requisition.

Tissue by tissue, our physical organization has been patiently and toilsomely unravelled—its functional operations, both in health and disease, and the laws that govern them, attentively studied; and though neither an exemption from, nor a perfect contral over, this common enemy to our race, has been obtained, the inquiry has not proved fruitless. But, great as have been the triumphs of medicine, there still remains much to be learned. Research, experience, and observation, will continue to elicit new truths, and throw additional light upon every department of the healing art.

This is essential; for, operated upon as we are, by a multitude of surrounding influences and causes, our constitutional temperaments and tendencies, are constantly changing, and, consequently our liability to particular forms or kinds of disease, is correspondingly increased or diminished.

These changes have been, and will continue to be noted. Fact upon fact, will accumulate, and as past errors and false notions shall be exploded, correcter views, both in theory and practice, will be established. From year to year, will the knowledge of disease, and the means of its prevention and cure, be more and more augmented; and, while, by reason of man's fallen nature, we can never hope to banish it from the world; it is no more than the part of sound philosophy, to expect that an increased mastery over it will continue to be acquired. The science is progressive; its advance is steady, though every year does not produce an Hippocrates, a Galen, a Celsus, a Paré, a Fauchard, a Hunter, a Jenner, a Blake, a Fox, or a Rush; its course is, nevertheless, onward; and while every now and then a mighty genius appears, to astonish the world with his dazling and gigantic powers of intellect,—seeming to obscure, for a time, the rays of all lesser lights, it has numbered among its cultivators hundreds of men of a high order of talent, who have laboured in the cause with no less zeal; and to them its present state of perfection is, in an eminent degree, attributable. At the present time, both the dental and medical professions are adorned with a greater array of talent than they ever were at any former period.

Though charlatanism, in all the departments of the healing art, as in days of greater ignorance, continues, with unblushing impudence, and voracious appetite to stalk abroad in open day, and prey upon the pockets of the credulous, the advocates of scientific truth and the real lovers of mankind are becoming more and more numerous, and with ever fresh, accumulating and persevering zeal, are labouring to acquire a still better and more perfect knowledge of the phenomena, and cure of the multitudinous maladies incident to the organism of man. As the fruits of their labour, every day proclaims some new triumph, either in one or other of the branches of medicine. In surgery, operations are now almost hourly and successfully performed, that were not even thought of ten years ago. Congenital deformities that were then regarded as irremediable, are now, by the mere division of a muscle or tendon, with the greatest ease and safety removed.

But, while surgery has thus been reaping rich and imperishable laurels, the other branches of medicine have not lagged behind. They, too, have achieved new victories—acquired fresh honors. The recent improvements in general pathology and therapeutical agents, have contributed, in the treatment of disease, to the most happy results. Unlike, however, the brazen-faced and selfish harlequin, who, for fear of exposing his empty pretensions, keeps whatever meagre knowledge he may, perchance, possess, closely locked within his own breast, the true cultivators of the science, no sooner make a discovery which they deem of value to the well-being of their fellows, than they promulgate it to the profession, and to the world. For instance,—had Jenner, the discoverer of the vaccine virus, withheld the secret from the world, he might have commanded a large part of the wealth of the whole globe. The discovery was one in which the welfare of the whole human family were most deeply interested; affording, as it does, protection from one of the most loathsome and destructive maladies that was ever visited upon mankind; and he, with the most noble and praiseworthy philanthropy, chose, rather than to enrich himself in that way, to remain in comparative poverty. It is to the promulgation of the discoveries that have been made, and the publicity that has been given to the results of the experience and observation of those who have occupied themselves with the calling, that the art of medicine is enabled to alleviate so much of human suffering. Concealment is the secret of the success of empiricism, but the man of genuine worth fears not to submit his pretensions to rigid scrutiny. Based upon scientific truths and sound philosophy, he does not fear to have them examined, nor does he grudge others the knowledge which he possesses, but rather seeks to disseminate it as widely as possible. Facilities for its acquisition are placed within the reach of almost every one: elaborate works on the various branches of the science, and periodicals devoted to it are daily issued from the press. Public institutions too, for instruction in it have been established in almost every part of the world, so that, while the exercise of the duties of the physician may be said to be confided to only a few, nearly all may, to a certain degree, acquaint themselves with, at least, the general principles of the art.

Thus publicly has medical inquiry been conducted, its results made known, and thus has the science flourished; but while most of the branches of this noble profession have been fostered by learned associations and legislative enactments, there is, at least, one, which has not been so highly favoured. It has had greater difficulties to contend with, and if it has in any degree kept pace with the others, it is because of the exertions of a few, who have applied themselves to its culture with a perseverance that has never faltered. I allude to dental surgery. This branch of medicine, though it has heretofore attracted much less attention than any of the others, has nevertheless, attained, to a very high state of excellence, and it is beginning to assume a greater importance; its value is becoming better known, and the same means are now being employed for its more rapid advancement, that have contributed so largely to the perfection to which the other branches of the curative art have been brought. The formation of the society which I now have the honour to address, and at whose instance these remarks are made, may be mentioned as one, and not among the least of the means that have been resorted to for the accomplishment of this desirable object; and that it will contribute very largely to it, if its designs be fully carried out, no one can hesitate to affirm. But the efforts of this association do not constitute the only means that are at this time in operation for the improvement of this branch of the science, others equally valuable and effective are in operation; but of these it is not my purpose to speak at this time; yet I cannot allow the occasion to pass, without briefly adverting to the information with which we are at present possessed, and in doing this, it will be necessary to refer to the progress and present condition of the literature of the art.

With the early history of the progress and condition of the art of the dentist, we know but little, so that whatever knowledge of it, is at present possessed, it is not an inheritance from antiquity, but is the result of modern research, inquiry, and observation. We know that it was practised in ancient Egypt, and *Hippocrates*, who is styled the father of medicine, notices the dentition and some of the diseases of the teeth, and, we are told by Ambrose Paré, a celebrated French surgeon, that he even described the method of inserting artificial teeth. That this operation was common in Rome anterior to the christian era, we have conclusive evidence. But such of the writings of those of the remoter ages, whose attention was directed to the dental organism, as have survived the wreck of time, and are now to be had, only go to show, that their knowledge of the teeth, their disease, and prosthesis was exceedingly crude and imperfect. To notice any of their opinions then is unnecessary, and more especially so, as they do not accord with the generally received doctrines of the present day. Although dental anatomy, pathology, and therapeutics, had made some progress previously to the eighteenth century, it was not until about the commencement of this, that the art of the dentist attracted much attention, or assumed a distinctive character from among the other branches of medicine.

In 1728, Fauchard published his famous treatise, entitled *Le Chirurgien Dentiste,* and notwithstanding the difficulties under which he must have laboured in the procurement of materials for his work, in consequence of the paucity of the knowledge of the period at which he wrote, he has treated, and that too at greater length than almost any subsequent writer, on nearly every subject necessary to be embraced within the professional education of the dental surgeon. It is comprised in two 12mo. volumes, making altogether upwards of nine hundred closely printed pages, and though more recent research and experience has shown many of the theoretical opinions of the author to be incorrect, and greatly improved upon the plan of practice pursued by him in the treatment of the maladies of the mouth, yet it cannot be read without profit, or fail to command admiration. It is the result of extensive and laborious research and long and attentive observation, and contributed in an eminent degree, by the spirit of inquiry that it awakened, to the perfection to which the art has at present attained, and justly, therefore, has the author been styled the "*father of modern dental surgery.*"

Besides this work, many others appeared, not only in France, but also in England, Germany, and other countries during the eighteenth century; and, among the most distinguished of the writers on adontology of this period, the names of Bunon, Bourdet, Jourdain, Berdmore, Hunter, and Blake, may be mentioned. There might also be added to this list upwards of a hundred and twenty others, but previously to the commencement of the nineteenth century, the treatment of the diseases of the maxillary organs was not very well understood, nor had the manner of substituting artificial, to supply the loss of the natural teeth, attained to much perfection. The works, therefore, that issued from the press anterior to this time, while they threw much valuable light on the anatomy and pathology of the dental apparatus, contained but little correct practical information; but, as the knowledge of the structure and diseases of the teeth advanced, more rational methods of treatment began gradually to be adopted, and in the space of a few years, a greater and more thorough control was had over the maladies of these organs, than has ever been possessed over those of any of the other parts of the body.

With the commencement of the present century a new era began to dawn upon the dentist's science. The inaugural dissertation of Robert Blake, on the structure of the teeth of man and other animals, published in 1798, was but the precursor of a host of other works on all the parts of the dental art. The origin, formation, structure and dentition of the teeth, the means for their preservation, and the prevention and cure of their diseases, together with the best methods of supplying their loss, began from this time to be studied with increased interest and attention. Every department of the subject was submitted to more thorough investigation, and the research has been continued with unabated zeal down to the present time.

As in the earlier history of dental surgery, so still the French continue to be its most zealous cultivators. In 1800, Professor *Baum,* published a very elaborate treatise on first dentition and the diseases that accompany it. This was a prize essay, and while, perhaps, it is the best work that has ever appeared on the subject on which it treats, it is far from being faultless. *Laforque,* published a work on the theory and practice of the art of the dentist in 1802, which contains some correct practical information, but like Professor Baum's on first dentition, it is not wholly free from errors. It is, however, the best work that had appeared at the time of its publication. The same author published in 1806, a treatise on the semiology of the mouth, which I shall have occasion hereafter to refer to more at large, and in 1808, a series of articles on the diseases of the teeth, and in 1809, a dissertation on first dentition. He was an attentive and for the most part, a very accurate observer.

*Duval,* is also an author of considerable eminence. In addition to being an acccomplished and elegant writer, he was thoroughly read in dental science. In 1802, he published an essay on the extraction of the teeth, or rather on the accidents consequent upon that operation; in 1803, he published another on odontalgia, and in 1805, a very interesting work entitled "Le dentiste de la jeunesse." Prefacing this last, is a paper entitled "Councils of the Ancient Poets," on the preservation of the teeth, in which no little research and ingenuity is displayed. In 1820, he published a small work on second dentition, and in 1828, a treatise on mechanical dentistry. Besides these, he is the author of several other well written papers on the teeth.

In the same year (1805,) in which Duval's "Dentiste de la jeunesse," appeared, *Gariot,* published a treatise on the diseases of the mouth, and in the year following, *Leroy* (*de la Faudiguere*) published a work on the diseases of the gums.

In 1807, a work entitled "The Art of the Dentist," by *Maggiolo,* was issued from the press. This treatise was quite popular in its day, and is now held in some estimation as a work of reference.

The next work which I shall mention, is entitled "Odontology," and is from the pen of that accomplished dentist, *C. F. Delebarre.* This work abounds with much valuable and correct information, but is not free from faults. It was published in 1815. The same author published in 1819, his celebrated treatise on second dentition, which is probably one of the best works on that subject that has ever appeared, and besides, treating fully and at length on the dentition of the adult teeth, it has one chapter on salivary calculus, and one on the semiology of the mouth. The same author, the year following, (1820,) published a treatise on mechanical dentistry, illustrated with forty-two plates, and in 1826, a supplement to his treatise on second dentition, a work of upwards of eighty octavo pages. To the pen of Delebarre, the literature of dental surgery is probably as largely indebted, as to that of any other individual, and while many of the views which he advances are susceptible of easy refutation, his opinions are for the most part correct.

*Lemaire*, is also an author of some note. He published in 1816, a manual on the anatomy and physiology of the teeth, and in 1822, a treatise on dental physiology and pathology.

*Serres*, an able and ingenious writer, published a treatise on the anatomy, physiology, and dentition of the teeth in 1819, that has attracted considerable attention. Many of his physiological opinions have been severely animadverted upon, and the justness of his claims to anatomical discoveries which he pretends to have made, questioned.

The improvements in mechanical dentistry have fully kept pace with those in the other departments of the profession, and not among the least of these is the manufacture of incorruptible artificial teeth from a combination of earthy and mineral substances. Of this invention, as well as that of many others in the art, the French are entitled to the credit. Besides several able and well written papers that appeared on this subject in the early part of the present century, *Audibran*, published in 1821, a very full treatise upon it. These teeth, however, have been brought to greater perfection in America than any other country.

In 1825, *F. Cuvier*, published a treatise upon the teeth of mammiferous animals considered in their zoological characters, with one hundred plates. To the surgeon dentist, this work is interesting only in a physiological point of view.

*Meil*, published a work of considerable merit in 1826, on the direction of the secondary teeth, and in 1828, *Rousseau,* published his famous treatise on the comparative anatomy of the teeth of man and other animals. This is probably one of the best works of the kind extant. It is well written, and characterized by great depth of research; and previously to its appearance, in 1820, the same author published a dissertation on first and second dentition.

In 1828, also, *Maury,* published an excellent treatise on all the parts of the art of the dentist, illustrated with forty plates. Two other works by the same author appeared several years before this.

A very valuable treatise on dental anatomy by Professor Blandin, was published in 1836. This work, though not large, deserves to be ranked among the first upon the subject on which it treats.

To the foregoing enumeration of works on the teeth, twice as many more might be added, but the mention of these will suffice to show the range which the study of odontology has taken in France since the ushering in of the present century.

Leaving the French school, it may be well to examine the progress which the science has made during the same time in Great Britain; and if I mistake not, the inquiry will show that, although its advance in France has been rapid, it has been little less so there. As early as the year 1803, the first part of *Fox's* celebrated treatise on the "Natural History and Diseases of the Human Teeth," appeared, which, in 1806, was followed by the second part. This work has held a justly deserved high place in the literature of dental surgery. It has been quoted from by almost every subsequent writer, and on the anatomy and physiology of the teeth has been improved upon but very little. On the diseases of the teeth, while the doctrine set forth by the distinguished author is maintained by several very able European writers, it is denied by others of equal acumen and ability. Though its advocates have displayed no little talent and ingenuity in its support, its opponents have adduced arguments and facts against it, that would seem to set all doubt upon the subject at rest. Therapeutical dentistry was not very well understood at the time of the publication of this work, it contains, therefore, but little information of value on the treatment of the teeth. Of the estimation, however, in which this work is held, some idea may be formed from the fact that it was translated into French in 1821, by Lemaire.

Four years after the publication of Mr. Fox's work, a treatise by *Fuller*, on the structure, formation and management of the teeth was issued from the press, and the year following, a volume by *Joseph Murphy*, entitled, "Natural History of the Human Teeth," with a treatise on their diseases from infancy to old age, was published. These works, though better suited to the general reader, than the professional dentist, are not to the latter devoid of interest. In 1819, a treatise by Mr. *Bew*, appeared, and in 1823, Gerboux, published a work on the diseases of the teeth.

But one of the best works that has issued from the English press on dental pathology and therapeutics, is from the pen of that able and accomplished practitioner, *L. Koecker*, M. D., and is entitled, "Principles of Dental Surgery." Though perhaps the author may have incurred the displeasure of a few, by the free manner in which he denounces empiricism, his work is nevertheless valuable, and has doubtless contributed in no small degree, to the success that attends the present mode of practice in the treatment of maxillary diseases. In 1828, he published a work on the diseases of the jaws, and in 1835, an essay on "Artificial teeth, obturators and palates."

Mr. *Fay* published in 1827, a work describing the mode of using forceps invented by himself for the extraction and excision of teeth. The advantages proposed by this last operation have not been realized. For obvious reasons, it has become necessary to abandon the practice. In 1828, *J. Patterson Clark* published a small treatise on the diseases of the teeth,—advocating the doctrine that dental caries is the result of the action of external corrosive agents.

The next work which I shall mention, is one that is very favourably and extensively known. I allude to the treatise on the anatomy, physiology and diseases of the teeth, by Mr. *Thomas Bell*, an able and highly accomplished writer, which was published in 1830. A work on operative dentistry, of considerable merit, by Mr. *Snell*, was published in 1831, and in 1835, Mr. *Robertson* published an excellent treatise on the diseases of the teeth; in which he maintains the theory that their decay is attributable to the decomposing action of external solvents.

Mr. *Alexander Nasmyth*, a gentleman of unquestionable ability, and a fine writer, has recently published an historical introduction to a work which has not yet appeared, on the "development, structure, and diseases of the teeth." What the author's views are upon these subjects, are not made known in the part of his work now from the press. That his treatise will contribute very much to enrich the literature of dental science, no one can hesitate to affirm. Curiosity has at least been awakened by the part that has already appeared, and the remainder is looked for with no little impatience.

The last English work which I shall mention as having been published within the last forty years, is from the pen of Mr. *Jobson*, and treats upon the anatomy, physiology and diseases of the teeth. It was published in 1835, and is a work of some merit. To this list many others might be added. It will be seen, however, from those already mentioned, that the progress of dental surgery in England, since the year 1800, has nearly, if not quite, kept pace with the advance of the science upon the continent.

In Germany its progress has been less rapid, yet it has there attracted considerable attention; and from that country a number of works have emanated.

The researches of Professor *Retzius*, of Sweden, have excited much attention in Europe, and though they do not go to confirm previous opinions in regard to the structure of the teeth, they have nevertheless thrown much light upon the subject. They were conducted upon an extensive scale, consisting of microscopic examinations of the teeth of man, and those of a great many other animals, and go to prove the structure of these organs to be tubular. A translation of the account of his microscopical researches may be found in Nasmyth's "Historical Introduction" to his work on the "development and structure of the teeth." An account of the researches on the same subject of *Purkinje* and *Müller*, is also contained in the same interesting work.

In America, and especially in the United States, though there has not been as much written on the science of the teeth as in Europe, and notwithstanding the charlatanism of the profession in this country, its advance has not been less rapid; and, there is not perhaps any country in the world, where the duties of the surgeon dentist are more successfully exercised. I might also mention a number of very valuable works and papers that have emanated from American dentists, and which, it may be, would not suffer by comparison with many of the best European dental publications; but having already extended this part of my subject to a greater length, than I had at first intended, it may not be well to refer to them in detail, and besides, the occasion is not, perhaps, the most proper. Let it suffice to say that efforts more vigorous and better calculated for the improvement of the art, than those which are in progress here, are not in action in any part of Europe.

From the foregoing brief and imperfect expose of the progress of this department of science, some idea may be formed of its present condition, and of the extent of the range of inquiry upon it; and from this it will be seen, that it, as well as the other branches of medicine, has been a subject of much investigation. For the last half century, at least, its advance has been steady, and at no previous period of its history, has it been studied with more persevering industry than at present.

But notwithstanding the perfection to which it has been brought, and the inquiry that is now abroad upon the subject, I cannot but believe, that the success of the dental practitioner, in the treatment of the diseases committed to his care, would be greatly increased, by a more thorough knowledge than is generally possessed of the physical characteristics of the several parts of the mouth. The general practitioner might also profit by the indications that are to be found in the appearances that are here exhibited.

Regarding a knowledge of the physical characteristics of the mouth then, as of greater importance than is usually supposed, the remarks which I shall submit to the consideration of the "American College of Dental Surgeons," on the present occasion, will be principally confined to this subject, and not so much that they will supply any *hiatus* that may exist in American dental or medical literature, as that they may be instrumental in awakening inquiry upon it, and in inducing some one more competent than myself to take it up, and treat upon it more at large.

Upon the physical characteristics of the mouth, little has been said by American or English dental or medical writers; and for whatever information the author of the following inquiry may possess upon the subject, apart from what he has gathered from his own observations, he is principally indebted to French authors. From the writings of Laforque, Delabarre, and Mahon, he has derived much benefit; and to them, though he does not concur in all the opinions which they have advanced, he takes pleasure in acknowledging his indebtedness. The most of the pathognomic signs which he has mentioned as belonging to the tongue have been described, together with many others, by professor Schill, in his treatise on semeiology.

### GENERAL CONSIDERATIONS.

The susceptibility of the physical organization of man, to morbid impressions, differs in different individuals. In some, its functional operations are liable to be deranged from very trifling causes, while in others, they are much less easily disturbed. Nor do the same causes always produce the same effects. The susceptibility and tendency of the organism or part upon which they act, determines their character; and this is true, both with regard to constitutional and local diseases: with the physical structure generally, and all its parts separately considered, but with none more so than the teeth, gums, and alveolar processes. The teeth of some persons are so susceptible to the action of deleterious agents, that they no sooner emerge from the jaws, than they become involved in general and rapid decay, while those of others, though exposed to the same causes, remain unaffected through life. A similar difference of susceptibility exists, also, in the parts within which these organs are contained.

With the teeth, these differences of susceptibility, to be morbidly affected, are implanted in them at the time of their formation, and are the result of the different degrees of perfection with which this process is accomplished; and, as I have remarked on another occasion,* I repeat, in proportion as these organs are perfect, their capability of resisting morbid impressions is increased, and as they are otherwise, it is diminished. This is true of every part of our being; but as the teeth are formed, so they continue, except they be impaired by disease. We must qualify this remark, how-

* See my Treatise on Dental Surgery.

ever, by observing that they gradually acquire a very slight increase of density, whereby their liability to this is correspondingly lessened.

Not so, however, with the other parts of our physical structure. They may be innately delicate, or imperfectly developed, and afterwards become firm and strong, or be at first healthy and well-formed, and subsequently become impaired, and, in proportion as they undergo these changes, their susceptibility or tendency to disease is increased or diminished. But the teeth are not governed by the same laws, neither physical nor vital, that regulate the operations of the other parts of the animal economy. Not only is the manner of their formation, but their diseases also, are different. The other tissues of the body, not excepting those which are identical in structure with these, are endowed with recuperative powers, whereby an injury sustained by them may be repaired by their own inherent energies, but the teeth do not possess such attributes.

Assuming these propositions then to be true, and that they are, especially those with regard to the teeth, I shall endeavour to make appear, it becomes an object of no trifling importance to discover the signs, if there be any, (and that there are, I think, I shall be able to show,) by which the susceptibility or tendency of the human organization to disease, may be determined. But to do this, except in so far as the teeth, gums and alveolar processes are concerned, is not the object of the present inquiry, yet in the prosecution of the task that has been imposed upon me, I may take occasion to advert to certain constitutional, and other local tendencies that are indicated by the appearances and condition of the dental apparatus, the other parts of the mouth and its fluids.

M. Delabarre, affirms that by an inspection of the teeth, we can ascertain whether the innate constitution is good or bad, and my own observations go to confirm the truth of this opinion; but as this author adds a little further on, these are not the only organs that should be interrogated. The lips, the gums, the tongue and the fluids of the mouth should also be examined, to discover the health of the organism, and ascertain whether the original condition of the constitution has undergone any change.

Those who have not been in the constant habit of closely observing the appearances that are met with in the mouth, may be somewhat sceptical in regard to the information derivable from them concerning the constitutional health, but those who have studied them with care, will not hesitate to assert, that they are, in very many instances, by far more certain and accurate, than any which can be obtained from any other signs. For example—the periods of the ossification of the different classes of both sets of teeth being known, we are enabled to discover whether the original or innate constitution was good or bad by the physical condition of these organs, for as the functions of the organism were at this time healthily or unhealthily performed, they will be perfect or imperfect; or in other words, their texture will be hard or soft.

In a treatise, entitled "*De dentitione,*" attributed to Hippocrates, but with how much truth I am unable to determine, it is said, three periods of ossification are ascribed to the teeth—the same also, is acknowledged by M. Baumes. The first is while the fœtus is in the matrix, and has reference only to the temporary teeth, which are in part ossified at birth. The second generation or period of ossification, is confined to those which are formed during lactation, and consists, according (as M. Delabarre says,) to the "father of medicine," of the incisores and cuspidati of replacement, and the first permanent molares. The third period embraces those which are formed from solid aliment, and consists of the second and third molares. The physical condition of the teeth will enable us to determine with an accuracy that can be relied on, the state of the constitutional health at the time they were respectively being formed.

Although, as has often been remarked by writers on odontology, the teeth of the child, like other parts of the body are apt to resemble those of its parents, so that when those of the father or mother are bad or irregularly arranged, a similar imperfection will generally be found to exist in those of the offspring, it does not necessarily follow, and when it does, it is the result of the transmission of some constitutional impairment, whereby the formative process of these small bones is either disturbed or prevented from being effected in a perfect and healthy manner. The teeth of the child, therefore, as an eminent French writer,* correctly observes, may be said to depend upon the health of the

* Delabarre.

mother, and the aliment from which it derives its subsistence. If the mother be healthy, and the nourishment that is given the child be of a good quality, the teeth will be dense and compact in their texture, generally well formed and well arranged, and as a consequence less liable to be acted on by morbid impressions, than those of children deriving their being from unhealthy mothers, or that subsist upon aliment of a bad quality. Temperament, also, as will hereafter be made to appear, exercises no mean influence upon the functional operations of the body. Upon it, the constitutional health depends to a greater extent than pathologists generally admit, and hence it is, that that of the child usually partakes of that of one or other, or both of its parents. "This," says M. Delabarre, "is particularly observable in subjects that have been suckled by a mother or nurse whose temperament was similar to theirs." To obviate the entailment of this evil, he recommends mothers having teeth constitutionally bad to abstain from suckling, and that this highly important office be entrusted to a nurse having good teeth,—asserting at the same time, that, by this means, the transmission of so troublesome a heritage as a bad denture, may be avoided.

Depending, then, as, not only the physical condition of the teeth, but that also, of the organization generally, confessedly does, upon the quality of the nourishment from which subsistence is derived during infancy and childhood, it is highly essential that this be good, and that that, especially, derived from the breast, be from those only who are in the enjoyment of health, and possess good constitutions. Whenever, therefore, the employment of a wet nurse becomes necessary, the greatest care should be taken to secure one possessing these highly important and desirable pre-requisites; and this cannot always be done without a knowledge of the signs that are indicative of the state of the constitutional health and temperament; and, as M. Delabarre judiciously remarks, there are cases where the physician cannot be too well assured whether the present good health be innate, or whether it is acquired,—stating at the same time, as an example, that "when a strange nurse is about to be employed, he cannot be too scrupulous." He also tells us that the inspection of the breast only enables us to judge of the development of the gland, and the quality of the milk as it then is, which may be good to-day, and in a short time become bad.

The truth of this observation has been verified in but too many instances, and it therefore becomes a matter of much consequence to find some signs upon which a greater reliance can be placed, than those which are to be found in the appearance of the breast, the cheek, or the beating of the pulse. These are often deceptive.

The following are the signs enumerated by professor Baumes, as essential for a nurse to possess. "An excellent nurse," says he, "should be of good morals; and, indeed, of fine physical qualities. Her age ought to be between twenty and thirty, and the colour of her skin natural. Her eyes should be lively and animated; her hair and her eyebrows brown or light coloured, her lips red, her teeth sound and good, her gums hard, and well coloured. She should have sweet breath; the nose unobstructed, and exhaling no odour—the neck sufficiently long, the chest large and well arched; her breasts ought to be loose, firm, and distended; elastic, and moderately large; with the nipples sufficiently irritable to be firm when the finger is passed under them; brown, long, and thin;—placed upon the middle of the declining part of the breast, in the middle of an elevated areola of an obscure, red colour."* Besides these, he describes the qualities that the milk should possess, and the tests necessary to their ascertainment.

These qualities are all essential, and though they at first be possessed, they do not furnish us positive assurance that there is no hereditary constitutional vice lurking in the system, that may not, by the debilitating process of lactation, become developed.

M. Delabarre, who seems to have devoted much time to the study of constitutional temperament, in remarking upon this subject, says, "Having been often consulted in the choice of a nurse, I have always paid much attention to the constitutional condition of the mouth, and whenever a child, born of weak parents, or of those having delicate teeth, has been confided to a good nurse, she has procured for it good teeth, and a good temperament, unless some severe disease has rendered useless this precaution."

Alluding to the fallibility of the signs usually relied upon as furnishing the requisite indications in this matter, the author, from whom I have just quoted, thus observes—"the semeiology of the

* Quoted from professor Thomas E. Bond's Translation.

mouth alone can make known to the physician whether that woman owes the beautiful carnation she possesses to her parents, or has attained it from a skilful regimen." "I have collected," says he, "some valuable facts on this subject, and I have met with some women, who, for a long time, have had a great interest in keeping out of view, that they had not always enjoyed as good health as they seemed to possess at the moment when they were soliciting employment. I would not have it thought that I suppose every woman having good teeth, is, indubitably a good nurse, for, even the most perfect constitution is susceptible of being deteriorated. We see, therefore, that to judge correctly of the past and present state of health, it is requisite to collect all the signs that can lead to this knowledge, and to obtain it, it is also requisite to be for a considerable time practised in these sorts of researches; for, as a beautiful carnation may mislead the judgment, so may handsome teeth be mistaken for good ones, which is not always the case. This remark is very important in practice, inasmuch as an infant suckled by a woman having bad teeth, very often has these organs similar to her's. These truths I could establish by a multitude of proofs, but they are so apparent, that it seems unnecessary to cite them."

Before I dismiss this part of my subject, I will quote from Professor Baumes, an anecdote, which he relates, conveying a no less severe than just reproof to mothers, who, regardless of the well-being of their offspring, commit those offices that nature designed them to perform, to mercenary nurses. "They who do so," he says, "merit the humiliation endured by the mother of the natural brother of Gracchus. This young Roman when he returned from a military expedition, brought to his nurse more magnificent presents than he did to her who had given him birth. My mother! he says, you carried me nine months in your bosom, but as soon as you saw me you abandoned me. My nurse received me gladly; she carried me in her arms, and nourished me with her milk for three years;—all she did was voluntarily done. You carried me in your bosom and nourished me with your blood from natural necessity. I feel more indebted to my nurse than to you, and I wished to show this by the difference of my presents."*

* Vide, translation by Professor Bond.

There exists some diversity of opinion in regard to the influence that the quality of the nourishment from which subsistence is derived during infancy and childhood, exerts upon the future health and constitutional temperament. We are told by M. Delabarre, that a child, though it derives its being from weakly parents, may, by proper regimen, acquire a good constitution and temperament, and the truth of this observation has been verified in many instances. Were it necessary, I could adduce several that have fallen under my own immediate notice. M. Mahon, a French dentist, and author of considerable acumen and celebrity, gives it as his opinion, that a person cannot be born with a good constitution, except those from whom he derives his being, be in good health, and of that age when life is vigorous. He at the same time admits, that a child coming from parents of the most perfect health, may have its constitution deteriorated by an impure lactation; and that a child coming from weakly parents, may acquire a good constitution, though it will always bear about it certain signs of that which it had inherited,—and thence, he deduces, that it is possible to discover, by an examination of the teeth, any tendencies that may be lurking in the system. He has certainly studied the subject very attentively, and his remarks are worthy of consideration. If all which he says be not true, many of his observations, I think, are susceptible of proof.

In treating upon the physiognomical indications of the teeth, he says, "Does the child derive its life from parents that are unhealthy? The enamel of its milk teeth will be bad; the teeth themselves, will be surcharged with a bluish vapour, and in a short time, will be corrupted by a humid and putrifying caries. When the parents are only weakly or delicate, the enamel of the primary teeth will have a bluish appearance, there will be a tendency in them to a dry caries, which does not ordinarily make much progress, and seldom causes pain."

Again he observes, "It was only by a determination to notice very accurately the differences which I remarked on the teeth of numerous individuals, that I obtained these first truths. In the first instance, they were little more than mere conjectures, but by being daily increased, have now become diagnostics, about the certainty of which, I flatter myself, I cannot be deceived. It affords me pleasure to give an account in this place of a part of the means which I employed to arrive at the point which was the object of my researches. When I perceived some signs, as for example, shadowy lines on the primary teeth, and those of replacement of different children, I put all my application to work for the ascertainment of their cause, and when I believed I had found it, I interrogated their mothers, who generally confirmed the judgment I had formed. I then went on further; after calculations that seemed to me highly probable, I ventured to declare the period at which a great crisis or pain had happened, and in such a month of pregnancy; and I have had the satisfaction to find, that I had conjectured correctly. My expectations, based on the same procedure have been crowned with success in *adults*, whose teeth, by the simple examination of them, have disclosed to me an advantage no less valuable than the first, namely, that of generally being able to tell, whether they were born of strong, weak, or aged parents, and also, if the mother has had several children, whether they were among the last," &c.

That a person experienced in these researches, may, by an examination of the deciduous teeth, tell, whether the mother, during the latter periods of pregnancy, had enjoyed good or bad health, there is no question. But it is very doubtful whether much can be ascertained, by an inspection of the milk teeth, concerning the health of the mother previously to the time of the commencement of their ossification, for upon the manner in which this is effected, depends their appearance and physical condition. The density of a tooth may be told at a single glance by a practised observer, and it is this and its colour, that are principally influenced by the condition of the system during ossification. The shape of the teeth is determined by that of the jaws and pulps before the commencement of ossification. I am of opinion, therefore, that nothing positive, concerning the health of the mother during the first five or six months of pregnancy can be learned from an inspection of the teeth of either dentition. From an inspection of those of the second, no information whatever in relation to it can be derived, and if Mahon was fortunate enough in some instances to tell what it had been, at an earlier period than that which I have mentioned, his prognosis was founded upon nothing more than mere conjecture.

The teeth while in a pulpy or ganglionic state, partake of the health of the organization generally. As that is healthy and strong, or unhealthy and weak, so will the elementary principles of which they are then composed, be deteriorated or of a good quality, but after ossification has commenced, the parts ossified cease to be influenced by or obey the laws of the other parts of the body. If the general health be good at the time this process is going on, it will be evidenced in their density and colour; if bad, in the looseness of their texture, &c.

This is a subject to which I have paid considerable attention. I have for a long time been in the habit of carefully noting the differences in the appearance of the teeth of different individuals, and of both dentitions, and though I have been able to conjecture in some instances what had been the state of the mother's health during the first months of pregnancy, candour compels me to confess that, I have never been able to find any signs in the peculiarity of their shape, size, density, or arrangement, that indicated it. But from the moment that that part of the formative process of these organs commences, which is not influenced by subsequent changes in the general economy, certain peculiarities of appearance are impressed upon them that continue through life, and about the certainty of the indications of which, in regard to the general health, I think there can be no question.

In commenting upon the views which M. Mahon advances upon this subject, Delabarre says,* "if he had thrown the light of repeated dissections upon them, he would have acknowledged with Hunter, Blake, Mauro, Fox and Bunon, that the secondary teeth do not begin to ossify until about the sixteenth month after birth, so that the good or bad health of the parents at the time of conception, cannot in any way affect the teeth of replacement, which are not formed until after the child comes into the world."

But however vague and erroneous may be some of the opinions of Mahon, he has certainly advanced many that are correct, and from which hints have been derived that have formed the foundation of some very valuable contributions to the science of the semeiology of the teeth.

* Vide, *Semëitique Buccale,* p. 225.

Lavater was laughed at and ridiculed for his enthusiastic belief in physiognomy, but the descriptions which he gives, with a view to the illustration of his favourite science, of the physical conformation of the various parts of the face, head, and other portions of the organism of man, embrace signs, which if applied to the study of semeiology, could hardly fail to lead to important results. Had the education and pursuits of this good and extraordinary man, fitted him for the investigation of this department of medical science, and had he entered into it with the same persevering ardour and zeal that he did that of physiognomy, he would have erected for himself an equally enduring monument of fame, and would thus perhaps have contributed as much to the amelioration of the condition of his fellows, as he has done by his physiognomical researches. In fact, of the importance of this subject, he seems to have been fully aware; and, after acknowledging his ignorance, he says the physiognomical and pathognomical semeiotica of health and disease ought to be treated on by an experienced physician, stating at the same time that, from the few observations which he had made, it was not difficult to discover the diseases to which an individual in health is most liable. He regards physiognomical semeiotics, founded upon the nature and form of the body, as of great importance to the medical practitioner, that he might be enabled to say to an individual in health, you may expect this or that disease sometime in your life. Possessed of this knowledge, he would be able to prescribe the necessary preventives or precautions against such maladies as he was most liable to contract.

Among the signs which Lavater notes as indicative of the temperament, he enumerates the shape, size, and arrangement of the teeth, but from the physical characteristics of these organs, when considered separately from other parts of the mouth, we only learn what the innate constitution was; they cannot be relied upon as indices to the state of the health subsequently to the time of their ossification. Their own liability to disease, however, may be determined by their appearance, and with the signs, therefore, that are indicative of this, every dentist should be familiar, to enable him, when consulted in regard to the attention necessary to the preservation of these invaluable organs, to prescribe such precautionary measures as shall secure them against the attacks of disease.

With regard, also, to the information to be derived from an inspection of the teeth, concerning the innate constitution, it has been well remarked by Delabarre, that physicians may derive much advantage in pointing out the rules of domestic hygiene for the physical education of children; for, says this eminent dentist, "can he admit of but one mode? Has he not, then, the greatest interest, to be well assured of the innate constitution of each, for whom his advice is required, to enable him to recommend nutriment suited to the strength of its organs? Will he report only on a superficial examination of the face, its paleness, the colour of the skin, all of which are variable? Will he not regard the repletion, or leanness of the subject, the state of the pulse, &c.? Surely he will make good inductions from all these things; but, the minute examination of the mouth, will give him, beyond doubt, the means of confirming his judgment; for, besides what we already know of the teeth, the mucous membrane of this cavity, receives its colour from the blood, and varies according to the state of that fluid." This latter is a fact, that the observation of the dental surgeon has an opportunity of confirming, almost every day; and which, when taken in connection with the physical characteristics of the teeth, together with those of the salivary and mucous secretions of the mouth, constitute data, from which, both the innate and present state of the constitutional health, may be determined with accuracy and certainty.

The symptoms of actual disease, have been minutely and repeatedly described, but the physiognomical signs by which the susceptibility of the human organization to morbid influences is determined, and the kind of malady most liable to result therefrom, do not appear to be so well understood. "Whatever," says the author last quoted from, "may be the knowledge which a practitioner may acquire of the changes that a disease, or even a tendency to a disease, may effect in the use of the functions of some organs, it is, at least, advantageous to be able to conjecture what has happened, in the whole of the system at another time. In fact, can a physician, when about to prescribe for a slight indisposition of a person whom he hardly knows, rely entirely upon the sabulous state of the tongue? Does not its aspect singularly vary? Is it not notorious, that in certain persons it is always red, white, yellow, or blackish? I, as well as others, have had occasion to make these observations on persons with whom it was always thus, but without their being subject to any of those indispositions that are so common in the course of life." These signs are as variable in sickness as they are in health, and consequently, can only be relied upon as confirmatory of the correctness of other indications that manifest themselves in other parts of the body.

The physical changes produced by, and characteristic of disease, have been described, both by ancient and modern medical writers, but the works that have appeared upon this subject, do not comprise all that is necessary to be understood. For example,—if we examine the lips, tongue, and gums of a dozen or more individuals who are regarded as in health, differences in their appearance and condition will be found to exist. The lips of some will be red, soft, and thin; others red, thick, and of a firm texture;—some will be thin and pale, others red on the inside, and pale on the edges;—some are constantly bathed with the fluids of the mouth, others are dry, and these differences of appearance and condition, are as marked on the tongue and gums as they are upon the lips, and are supposed to be attributable to the preponderance or want of existence in sufficient quantity of some one or more of the elementary principles of the organization. Hence, may be said to result the differences in temperament and susceptibility of the body to the action of morbid excitants.

A German writer* of considerable distinction tells us, and the same also is asserted by others, that the body is composed after an established manner, "of various congruous and incongruous ingredients," and "that there is," to use the metaphor, "a particular recipe, or form of mixture, in the great dispensatory of God, for each individual, by which his quantity of life, his kind of sensation, his capacity and activity, are determined; and that, consequently, each body has its individual temperament, or peculiar degree of irritability. That the humid and the dry, the hot and

* Vide Lavater's remarks concerning temperaments.

the cold," continues he, "are the four principal qualities of the corporal ingredients, is as undeniable as that earth and water, fire and air, are themselves the four principal ingredients." "Hence," he argues, "that there will be four principal temperaments; the choleric, originating from the hot; the phlegmatic, from the moist; the sanguine, from air, and the melancholic, from earth. That is to say that these are predominant in, or incorpoted with the blood, nerves, and juices, and indeed in the latter, in the most subtle, and almost spiritually active form. But it is equally indubitable to me, that these four temperaments are so intermingled that innumerable others must arise, and that it is frequently difficult to discover which preponderates; especially since, from the combination and interchangeable attraction of those ingredients, a new power may originate, or be put in motion, the character of which may be entirely distinct from that of the two or three intermingling ingredients." The truth of these propositions will hardly be questioned, and their admission at once affords a satisfactory explanation of the differences in the susceptibility of different organisms to the attacks of disease.

Assenting to these truths, and they are so self-evident that none can doubt them, I think it may be safely assumed that, as is the quality and respective proportions of the materials furnished for the growth, reparation and maintenance of the several organs of the body, so they will be. If, as I have before remarked, these be good, and in proper proportion, they will be endowed with health and vigour, and, as a consequence, qualified to execute their respective functions in a healthy manner. But if their elementary ingredients, to use an expression of the author from whom I have just quoted, be bad, their functions will be more or less feebly performed.

These materials are furnished by the blood.* From this fluid,

* Of the various writers who have treated upon this fluid, Magendie ranks deservedly high. To his persevering and laborious researches we are indebted for much valuable information. Not satisfied with mere conjecture, he instituted a great variety of experiments upon animals, which go very conclusively to prove that, no one of its constituents can be dispensed with without manifest and serious injury to the whole organism,—and that, it is dependent for its living principles upon the motion that is given to it in the circulatory system.

each organ receives such as are necessary to its own particular organization. The blood, therefore, exercises a most important influence upon the whole of the mechanism of the body,—determining, as it most undoubtedly does, the state of the health of all its parts, which, as M. Delabarre observes, is relative to its quality, and "that the general health results from that of all the system." In order to this, harmony must exist between all the organs, but in consequence of the great variety and intermingling of temperaments, it rarely does, except, perhaps, in those people in whom the sanguine greatly predominates, and who have not become enervated by irregular and luxurious modes of living. And, even when it does exist, we are by no means certain that it will continue to do so; for exposed as the body is to a thousand causes of disease, its functional operations may at almost any moment become disturbed. Among the civilized nations of the earth, the peasantry of Great Britain probably possess as good constitutional temperaments as are any where to be found; and, yet, even with these people, we are told that, although the sanguinous greatly predominates in a large majority of the subjects, it is combined and intermingled, in a greater or less degree, with others.

In all of these modifications the blood plays an important part; it determines the temperament of the individual, and as a consequence, the physical condition of all of the tissues of the body that are subject to the general laws of the economy. But the dependency of the solids upon this fluid is only mutual; it also is dependent upon them, and the condition of the one is relative to that of the other. The solids, if I may be allowed to use the metaphor, are the distillery of the fluids, while they in their turn nourish, repair, and maintain the solids. A change then in the condition of one, is followed by a corresponding change in the condition of the other. If the blood that is produced, be of an impure quality, or any of the substances that enters into its composition exist in too great or too small quantity, it will fail to supply the solids with the materials necessary to the healthful performance of their functions, and, if not actual disease, a tendency to it will be the result. And again, the purity of the blood is dependent upon the manner in which the solids perform their offices. While, therefore, duly appreciating the importance of this fluid, and its existence in a pure state, to the general health of the economy, I cannot ascribe to it, regardless of the functions of the solids, a controlling influence over the organization.

To distinguish all the nice and varied shadings of temperament, or states of the constitutional health, by the physiognomical appearances of the body, is perhaps impossible, or can only be done with great difficulty, and by those who have been long exercised in their observance, but to discover that which predominates is not so hard a matter, and its indications are no where more palpably manifested than in the mouth. By an inspection of the several parts of this cavity, together with its fluids and the earthy matter found upon the teeth, I repeat, inductions may be made, not only with regard to the innate, but also, the present state of the constitutional health, serviceable, both to the dental and medical practitioner; and, in the further prosecution of this inquiry, I shall endeavour to point out some of the principal of the indications that are here met with,—the appearances by which they are distinguished, and to offer such other general reflections as the subject may from time to time seem to suggest.

### OF THE PHYSICAL CHARACTERISTICS OF THE TEETH.

Dental practitioners, or, at any rate, the more observing of them, have noted the marked differences that exist in the appearance of the teeth, the gums, lips, tongue and secretions of the mouth, of different individuals. They have also noted those of that earthy substance, (commonly called tartar) which is deposited in greater or less abundance on the teeth of every person, and though all may not have sought their etiology, many have had occasion to notice, at least, their local indications, and to profit from the information which they have thus been enabled to derive. They have not failed to observe that the volume, colour, length, and arrangement of the teeth vary, and that these are indicative of the susceptibility of the organs to disease.

But to proceed,—teeth in which the earthy salts or phosphate of lime exists in great abundance, are generally of a dull, or heavy white, of a medium size, short, with thick cutting edges, those of each class of uniform dimensions, very hard, and after the middle period of life, gradually assume a faint yellowish appearance. This description of teeth is most frequently met with in persons of a sanguinous temperament, or at least, those in whom this greatly predominates; they rarely decay, and are indicative, if not of *perfect* health, of a state bordering very closely on it at the time they were being ossified in the subjects in which they are found. They are occasionally possessed by persons of all nations and classes, but far more generally, by labouring people in healthy northern latitudes. Among the inhabitants of England, Ireland, and Scotland, and more especially the middle and poorer classes, they are very common. They are also very frequently met with in the northern parts of the United States, the Canadas, the mountainous districts of Mexico, and so far as I have had an opportunity of informing myself in France, Russia, Prussia, and Switzerland. Those who have them, generally enjoy excellent health, and are seldom troubled with dyspepsia or any of its concomitants. It is this kind of teeth, which Lavater says he has never met with, except in "good, acute, candid, honest men," and of whose possessors, it has been remarked, that, their stomachs are always willing to digest whatever their teeth are ready to masticate.

But, as it regards character or disposition of mind, it is, perhaps, more than questionable, whether any thing can be learned from the physiognomical appearances of the teeth, further than they may be influenced at the time of their ossification by the constitutional temperament or state of the general health, and though this may then be favourable to the production of such as possess the characteristics I have described, it may afterwards become so impaired as to retain but little of its original condition, while these organs by reason of their exemption from the laws that govern the functional operations of other parts of the body, preserve theirs. Those who possess the temperament, however, necessary to the production of such teeth, usually have all their organs well developed, and as a very common consequence, have open, frank and cheerful dispositions. It is probable, therefore, that, from having very frequently observed them in the possession of those who enjoy these happy qualities of mind, that this celebrated physiognomist was induced to regard them as an invariable accompaniment.

In confirmation of what I have before said in regard to the influence which the state of the general constitutional health at the time of the ossification of the teeth, exerts upon their susceptibility to morbid impressions, it is only necessary to mention the fact, well known and frequently alluded to, of the early decay of a single class, or a pair of a single class of teeth, in each jaw, while the rest, possessing the characteristics which I have just described, remain sound through life. Thus, whenever it happens, that a child of an excellent constitution is affected with any severe disease, the teeth that are at the time undergoing the process of ossification, are found, on their eruption, to differ from those which received their earthy material at another time, when the operations of the organism are healthily performed. Instead of being of a dull or heavy white, and having a smooth uniform surface, they have a sort of chalky aspect, or are faintly tinged with blue, and have rougher and less uniform surfaces. Teeth of this description are very susceptible to the action of corrosive agents, and as a consequence, rarely last long.

But, not willing to rest the correctness of these views upon mere hypothesis or vague conjecture, I have, in a great number of instances, where I have met with teeth thus varying in their physical condition and appearance, taken pains to inquire of those who had had an opportunity of knowing the state of the general health of the subjects at the different periods of the ossification of these bones, and in every case, where I have been able to procure the desired information, it has tended to the confirmation of the opinion which I have here advanced. Nor have I neglected to improve the many opportunities that have presented, in the course of a somewhat extended professional career, for making these observations.

Although the operations of the economy are so secretly carried on, that it is impossible to comprehend their mechanism fully, it is well ascertained, that the phenomena that result therefrom are influenced and modified by the manner in which they are performed. If they be deranged, the blood, from which the calcareous materials that form the basis of all the osseous tissues are derived, is deteriorated, and furnishes these earthy salts in less abundance and of an inferior quality. Hence, teeth that ossify when the system is under the influence of disease, do not possess the characteristics necessary to enable them to resist the assaults of the corrosive agents, to which all teeth are more or less exposed, and that rarely affect those that receive their ossific matter from pure blood.

The calcareous ingredients of these organs are furnished by the red part of this fluid, and the gelatine that is in them is derived from the white or serous part;—"whence," as M. Delabarre, remarks, "it results that the solidity of these bones vary according as the one or the other of these principles predominates," and the relative proportions of these, as I have endeavoured to show, are regulated by the state of the blood at the time the teeth are undergoing ossification. In healthy subjects the blood is composed of about four parts of crassamentum, or clot, which is the red part, and one of serum, but the relative proportions of these are not always the same. Disease tends to diminish the red, and to increase the white or serous part of it. Sometimes the serum forms more than one-half of this fluid, and as this abounds at the time of the solidification of the teeth, they will be soft in their texture, and liable to decay.

The views of *Professor Hayden*, in regard to the solidification of the teeth, are, that the process is the result of the operations of the laws of vitality through certain affinities, that the gelatine that is in them is furnished by the serum of the blood, and the calcareous molecules from the red part of it,—the former serving as a bed for the latter.

The researches of Duhamel go to prove that bones acquire solidity no faster than the parts which are about to ossify become charged with red blood. The experiments of Haller, are also confirmatory of this opinion. And, M. Delabarre, in remarking upon the ossification of the teeth, says, "the superficial layer of the pulp reddens before it ossifies, whilst all below is entirely white; soon another layer reddens, is ossified and then whitens, and so on, successively."

There is one circumstance, however, that appears to have escaped the notice of osteologists, that might seem to militate against the doctrine that the calcareous ingredients of bony structures are exclusively derived from the red part of the blood; and that is, the increase of density which the teeth continue through life very gradually to acquire, notwithstanding the prevailing opinion, that the fluid they circulate subsequent to their ossification, is not so much as even tinged with red. But, that these organs are capable of being injected with red blood, has been satisfactorily shown. I have, in many instances, seen teeth that were tinged with red, but lest any one should be sceptical upon the subject, I will quote the following from an extract of a letter, addressed by Dr. H. H. Hayden, to Professor Dunglison, published in the "*Encyclopediæ of Medicine*," and copied into the "*American Journal of Dental Science.*"

"In certain varieties of asphyxia," says Dr. H. "the appearances of the teeth are not only singular, but highly instructive, in a physiological point of view, especially. They are almost uniformly tinged red. If examined immediately after death, they present a deep pink hue; if sometime after, the tint is darker. The different shades of colour, however, will generally depend in a great measure upon the age of the person, and the violence of the death." A little further on he remarks, that his "attention was attracted to these and to many other appearances of the teeth about the year 1815;" and that since that time, he has "observed them in numerous instances, both in human teeth, and in those of the bullock." Again, in another place he remarks, "In a letter received from my much respected friend and professional confrere, the late Edward Hudson, Esq.," dated Philadelphia, Sept. 11, 1818, he observes:—"In the small box you will find eight very fine teeth, if they were not red. I want your opinion of what occasions this redness, premising that the teeth of all drowned persons are so that I have examined."

Now, if teeth are capable, under any circumstances, of being injected with red blood, is it unfair to presume, that a portion of the crassamentum or red part of this fluid may become so diluted with the thinner or watery parts of it, as to be forced with them into the vessels of these organs by the impetus which is ordinarily given to it by the circulatory apparatus? I am of the opinion that it is not, and that it is in this way that the increase of density which these organs require is to be accounted for. A sufficient quantity of earthy salts, I should suppose, might be conveyed to them through the vessels with which they are penetrated, to effect the very trifling and almost imperceptible increase of density they attain to, subsequently to their ossification. The hypothesis, I am aware, does not accord with the views of Hunter in regard to the organization of the teeth, nor with those of several modern European writers on odontology, who maintain that these organs are not endowed with vascularity, but the incorrectness of this doctrine has been shown beyond the power of successful refutation. The circumstance to which I have just alluded, of the injection of the teeth with red blood, at once settles this question, and renders its further discussion on the present occasion unnecessary.

Having digressed thus far, I shall now proceed to notice a description of teeth quite different from those which I first described. The following are their characteristics:—They have a bleached, or azure blue appearance, they are most commonly long, the incisors thin and narrow, the cuspidati usually round and pointed, the bicuspides and molares small in circumference and deeply indented upon their grinding surfaces.

Teeth possessed of these characteristics are generally very sensitive, easy to be acted upon by corrosive agents, and to the ravages of which, unless great attention be paid to their cleanliness, they usually fall early victims. They have a soft chalky texture; the phosphate of lime and other earthy salts which they contain, are small in quantity, and as it seems, of a bad quality.* The kind of caries to which they are peculiarly subject, is soft, white and humid. The calcareous components of such parts of them as are affected with this disease is wholly decomposed, so that nothing remains but the gelatine, which may be removed layer by layer, whereas, as these organs approach in appearance and physical peculiarities those which were first noticed, caries in

* The analysis of the teeth of different individuals exhibit very different results. The relative proportions of the constituents of which these organs are composed are regulated, as I have before stated, by the state of the health of the subject at the time of their formation. If this be good, their calcareous components will be abundant, and of excellent quality, and in proportion as it is otherwise, these will be small in quantity, and inferior in quality.

them, is drier, of a darker colour, and possessed of greater density.

Thus, the colour, consistence, and humidity of dental caries, may be said, in most instances, to be determined by the density of the teeth, in which it occurs. The colour of it, however, may be, and doubtless is, in some cases, influenced by other circumstances; but as to what these are, I profess myself ignorant. They may consist in some peculiar modification of the agents—upon the chemical action of which on the organs, the disease is dependent; but this is mere conjecture, and the solution of the question still remains for future investigation. Whether it will ever be settled to the satisfaction of all, the future only can determine.

Having thus incidentally adverted to the decay of the teeth, I shall take occasion, while upon the subject, to notice an error that has obtained, to a considerable extent among European writers on dental science, in regard to the nature and cause of the malady. It consists in attributing to the teeth the same diseases that are incident to other bone, and was first promulgated by Mr. Fox. Subsequently, the doctrine has been adopted, with some unimportant modifications, by Bell, Jobson, and a few other English authors. It has also found favour to some extent, in the French school; but we are told by some of the continental writers, that dental caries, besides exhibiting phenomena, that are common to the disease in other bone, presents some that are peculiar to itself.

But, respecting the nature and cause of this disease, as occurring in the teeth, I have expressed my views somewhat at length in another place.* For the present, then, it will be sufficient to state, that, to the doctrine that it is analagous to caries in other bone, and is the result of inflammation of the bony structure of the organs, I wholly dissent, it being opposed both to facts and sound reason. If it were true that inflammation was necessary to the disease, it would never occur except in living teeth, and it is notorious that dead teeth, are as liable to its attacks as living ones. It exhibits, too, in these, all the phenomena that are manifested by it in the others. This is true, not only with regard to

* See my Practical Treatise on Dental Surgery, and vol. 1 of American Journal of Dental Science.

teeth that have lost their vascular connection with the general system, and are still retained in the mouth, but also with those that are placed there by art, if fabricated from bone or ivory. The vitality of the teeth may influence the progress character of the decay, though I am not certain that it does, but it is not, by any means, essential to it.

If the decay of the teeth then, is not referable to inflammation in their bony structure, to what is it to be ascribed? The inference is, that it is the result of the action of chemical agents, and when we take into consideration that the fluids of the mouth, when in a morbid condition, are capable of decomposing their enamels, if not possessed of more than ordinary density, and that the disease frequently commences upon this outer covering, the conclusion is at once irresistible. A most remarkable case of this description of caries, (called by Mr. Hunter, "*decay by denudation,*") is mentioned by Dr. Eleazar Parmly, in his notes to this gentleman's treatise on the teeth, published in the American Journal of Dental Science, in which the labial faces of several natural teeth, that had been artificially placed in the mouth, were attacked by it.

It may however, be asked, if caries of the teeth be produced by the action of external corrosive agents, how is it that the disease sometimes commences within the bony structure of the organs, and makes considerable progress there, before any indications of its existence are observed externally? I answer, that it never does commence within the bony structure of the organs; its attacks are always upon their external surfaces, sometimes upon the enamel, but most frequently upon the bone within the indentations on the grinding faces of the bicuspides and molares, and on the sides of the teeth at the points where they come in contact with each other, and where this outer covering is frequently so fractured by the pressure that is exerted upon it, that the juices of the mouth find ready access to the subjacent osseous tissue. The destruction of the organs may be gradually going on here for months and even years without any notable signs of its existence; and the commencement of the malady in these places have lead many to suppose that it had its origin within their osseous structure.

But a still more absurd and ridiculous theory in regard to the cause of the disease is advanced by Mr. Charles Bew. He attributes it to the arrest of the circulation in the organs, "by the lateral pressure of the teeth against each other."*

The exposure of the teeth too, to sudden changes of temperature, as from heat to cold, or cold to heat, have been regarded almost from time immemorial as a cause of their decay, but no explanation of the manner by which it produced the disease was attempted, until the promulgation of the doctrine that it was the result of inflammation, when it was numbered among, only the *exciting causes.* The popular belief that cold is a cause of dental caries, is traced back to Hippocrates, who, in mentioning the parts of the body that are injuriously affected by it, includes the teeth.†

M. Delabarre in alluding to this affection observes: "Caries of the teeth is a disease of their tissue, and not a simple decomposition proper to inert bodies. As an ulcer in a soft part is easily healed in a healthy subject, so the cure of this malady is effected with ease in those of good constitutions. Caries, in individuals of different constitutions, cannot be of the same nature, because their teeth have not the same solidity. Thus, those that are largely charged with gelatine will be exposed to *odonto-malaxia*, whilst those in which the calcareous phosphate is present in large quantity, will not be attacked with it as long as they preserve this advantage. But absorption being effected in teeth as in other bones, (which their softening in the interior and below the enamel, demonstrates,) consequently it follows, unless some external agent may have had power to act on the osseous tissue, that we can estimate the qualities of the humours by the nature of the caries that is manifested in the teeth."

"The softening of the interior tissue can result only from a derangement of the central ganglion; and this is caused by the arrival at this body of arterial blood deprived of the properties requisite to maintain it in a healthy state.

* See Bew on the teeth, pages 23, 64-5.

† Frigidum inimicum ossibus, dentibus, nervis, cerebro, spinali medullæ: calidum vero utile. *Aph. sec. v.*—par. 18.

The incorrectness of the opinion advanced by M. Delabarre, that caries of the teeth consists in something more than a mere decomposition of their tissue has been shown in the fact, that dead teeth decay as readily as living ones, and that caries in them exhibits the same appearances that it does in those that are possessed of vitality; and between the manner of the arrest of the progress of this malady, and that of an ulcer in soft parts, there is no more sameness than there is between the two diseases. The cure in the one case is effected by the restorative powers of the body, whereas, in the other, it is accomplished by the aid of art,—the operations of the economy not contributing in the least to the restoration of the injury.

The reason of the difference in the nature of caries of the teeth in individuals of different constitutions, is, as M. Delabarre has justly remarked, because of the difference in the density of the organs, which is determined by the temperament or state of the general health at the time of their formation. I am compelled to differ with him in the opinion that the softening of teeth that are not well provided with "calcareous phosphate," is owing to the removal of this earthy material by the absorbents, and that the disease is identical with that which in other bones, is denominated *mollities-ossium.* The action of the solvent agents that are concerned in bringing about that condition of the teeth designated caries, is facilitated by the softness of the organs, and hence, the rapidity of decay in those that are of a very loose and spongy texture, and the rare occurrence of the malady in those that are endowed with great solidity. The decomposition too, of their earthy salts is the more perfect, in proportion as they exist in small quantity. An impairment of the arterial blood, resulting from a "disturbance of the central ganglion," or any other cause, can have no agency in the decomposition or softening of the osseous structure of the teeth, except it gives rise to a morbid condition of the salivary and mucous fluids of the mouth. The destruction of the interior parietes of a tooth never takes place while the pulp or central ganglion remains; but this sometimes inflames and separates, and gives rise to the formation of an acrid and very corrosive humor; and, it is to the presence of this, that the softening of the bone surrounding the dental cavity, is attributable.

It remains to be proven that the absorbents have any agency in the destruction of the osseous tissue of these organs. That they do, not a shadow of evidence has been adduced, but that they do not, is rendered more than probable by the fact, that it takes place in those that are devoid of vitality, and if it can be effected without them in the one case, we have no right to suppose that it is not in the other.

I have said that no evidence has been adduced to show that the absorbents were capable of breaking down and carrying off the bony ingredients of the teeth, but I should have noticed the case mentioned by Mr. Thomas Bell,* of the occurrence of what he supposed to be an abscess in the substance of a tooth. From the drawing, however, which he has given of the tooth, it is seen that while the cavity extends to a considerable distance into its bone, the enamel constitutes its outer parietes,—a circumstance, that leads me to believe, that it was occasioned by the decomposition of this part of the osseous structure of the organ by some acrimonious humor that found access to it through an opening in the enamel. Indeed, cases are often met with of caries in teeth that are very soft, and not unlike the one described by Mr. Bell, where the destruction of both the calcareous and animal materials is so thorough that only a very small quantity of soft, moist, and white matter is found in the cavities occasioned by the disease. I cannot, therefore, under the circumstances, but believe, that Mr. B., though for the most part a close and accurate observer, and withal an accomplished writer, mistook this description of caries, for what he has termed an abscess.

Having departed thus far from my main subject, with a view of correcting what I conceived to be some palpable errors in dental pathology, I shall now resume its consideration. The great liability of the kind of teeth last described to decay, has already been noticed, and it may be proper to observe, that they are frequently atrophied, or have upon their surfaces white or brown opaque spots. These vary in size and number. Several are sometimes found upon a single tooth, and in some instances every tooth in the mouth will be more or less marked with them.

* Vide Anat. Phys. and Diseases of the Teeth, page, 171.

It rarely happens, however, that more than two or four of a single denture are thus affected; and it is worthy of remark, that those most frequently marked by it, are the central incisors of the upper jaw, and the bicuspides of the lower. The incisors of the lower jaw are rarely marked with this most singular affection, yet, they are, by no means, exempt from its attacks. I have met with a number of cases where they were atrophied.*

This, however, is not the only kind of teeth that are marked with this affection. These spots are occasionally met with on teeth of every degree of density, shape, shade, and size; but they are, probably, more frequently seen on such as those last described, than any other, and besides, it often happens that they are affected with erosion on emerging from the gums, and sometimes so badly as to place both their restoration and preservation beyond the reach of art. This species of erosion, or that which takes place while the teeth are in their matrices, is caused by some morbid condition of the fluid within which they are there bathed, and is denominated congenital.

But to proceed—teeth like those under consideration, are indicative of a weakly, innate constitution,—of a temperament considerably removed from the sanguinous,—and of blood altogether too serous to furnish materials, such as are necessary for the building up of a strong and healthy organism. They are more common to females than males, though many of the latter have them. They are met with among people of all countries, but by far more frequently among those who reside in sickly, southern latitudes, and whose systems have become enervated by luxurious modes of living. Among the inhabitants of Great Britain, they are more rare than they are among the Americans, and those who have them seldom attain to a great age. Nevertheless, some, under the influence of a judicious regimen, and a salubrious climate, though

* These spots upon the enamel or surfaces of the teeth, are, doubtless, caused by the destruction of the bond of union between these apparently crystaline coverings and the subjacent bone, and that, too, during the solidification of the organs. M. Delabarre attributes them to the death of a greater or less number of the exhalents, with which he contends that the enamel is furnished. And, to erosion, an affection entirely different, M. Duval applies the name that this affection is designated by.

innately delicate, as has been the case with most of those who have this kind of teeth, do acquire a good constitution, and live to a great age, while the dental apparatus, less fortunate, except the most rigid and constant attention be paid to the use of the means necessary for its preservation, generally soon falls an early victim to the ravages of disease.

There is another description of teeth, though differing in many of their characteristics from those of which I have just been speaking, that are, nevertheless, not unlike them in their texture, and in their susceptibility to deleterious impressions. The crowns of these are much larger than those of teeth of the ordinary size; their faces are rough and irregular, with protuberances, rising, not only from the grinding faces of the bicuspides and molares; but also, not unfrequently from their sides, with correspondingly deep indentations. Their appearance is that of a muddy white. The crowns of the incisors of both jaws are broad, long and thick. The posterior or palatine surfaces of those of the superior maxillary are rough, and usually have a deep indentation in them. In the majority of cases, their arrangement is tolerably regular, though they are more or less inclined to project. Their alveolar ridges usually describe a broad circle, and evidence a development, which, though large, is, in many respects faulty. The excess in size, both here and in the teeth, seems to consist more of gelatine than calcareous phospate.

This description of teeth decay readily, and in some instances appear to set at defiance the resources of the dentist. They are liable to be attacked at almost every point, but more particularly in their indentations and on their sides which come in contact with each other.

I am acquainted with a family, consisting of seven or eight members, most of whom are adults, and all have this kind of teeth. The most thorough and constant attention has been paid by each, to the use of the most approved remedial means, and yet all have lost a greater or less number of their teeth. They are generally first attacked on their lateral surfaces and in their indentations, but neither their labial faces nor most prominent points are exempt from the disease. No sooner than its progress is arrested in one place or part, than it appears in another. I

have had occasion to fill a single tooth in as many as four, five, or six different places, and in this way, though my efforts at the preservation of a considerable number of them have proved abortive, I have been able to save some for a few years. But it is not necessary to particularize cases. Every practitioner has seen teeth like those of which I am now speaking, and is aware of the difficulty of their preservation.

It may be remarked, however, that the corrosive properties of the fluids of the mouth are sometimes so lessened by an amelioration of the constitution, that notwithstanding the great susceptibility of the teeth, of being acted upon by them, they may not for years, or until the general health relapses into its former, or some other unfavourable state, exert any very active or decidedly bad effects upon them. This has happened in several instances that have come under the author's own immediate observation, and it should be borne in mind, that the solvent qualities of these juices are influenced by the state of the constitutional health. So far as that can effect these fluids, and no farther, it may exert a beneficial or prejudicial action upon the teeth.

Persons who have teeth like those now under consideration, generally have what Laforgue calls, lymphatico-serous temperaments. Their blood is usually pale, and the fluids of the mouth, while they are poured out in great abundance, are for the most part exceedingly viscid. They do not have that white frothy appearance that is observable in those of healthy sanguinous individuals.

Laforgue enumerates among the Europeans, three classes, each differing from the others in the quality of the materials that compose their respective organisms, that can be distinguished by their teeth.* The first of these classes have pure blood, a good constitution, and as a consequence, their osseous tissues are compact, their flesh firm, and all of their fluids of a healthy and good quality. The second class have sanguino-serous temperaments, and the third lymphatico-serous.

The first of these classes, he tells us, have handsome teeth, well enamelled and of a cream colour. The molares of the first

* Vide *Séméiologie Buccale et Buccamancie*, pp. 3, 4, 5.

dentition, of the second class, and sometimes the secondary incisors and cuspidati, are eroded, and the third class, he says, have teeth that are very white, brittle, and that are easily affected with caries.

The kind of teeth which he assigns to the third class, though frequently possessed by those who belong to it, are oftener met with among those of the second. It is among those of this class that the teeth which I last described are most frequently found.

Continuing the subject, he says, "there are in each constitution two degrees that are distinguished by the quality of the materials that compose the teeth. The second degree of the first class, has not so much of the bony structure, and their enamels are thinner than in the first degree; neither are the flesh, the blood, or any of the fluids of so good a quality. Persons of the second class are not so healthy as those of the first. There is nothing to distinguish them, except that the one constitution is more perfect than the other.

"In the second constitution erosion is more strongly marked in the first than in the second degree, and it has its seat upon the incisors instead of the large molares of the first dentition. In the third constitution, the second degree is marked by the softness of the teeth, which are more subject to caries than the first degree. The quality of the blood, the flesh, fluids, and the health and strength of subjects of the second and third constitutions are always in proportion to the density of the osseous system."

The present state of the constitutional health is not, as M. Laforgue supposes, indicated by the appearances of the teeth. The health of the general system is liable to impairment from a thousand causes, and though it be such, as may be necessary to "*handsome*, *well enamelled*, and *cream coloured teeth*," at the time of their formation, it may afterwards become bad while these organs will retain their primitive characteristics. If the character of the original constitution is maintained, its condition may be ascertained by the appearances of the teeth. It is probable, therefore, that Laforgue had reference to those cases only where it had not undergone any change, and if so, his views are unquestionably correct.

The classes of constitutions which he particularizes as distinguishable among the Europeans, by the appearances of the teeth are all met with in America, together with an almost countless number of intermediary ones. They cannot, however, be so readily determined from an examination of the dental organism, for the reason, that both present a much greater variety, and that though the first be primitively good, it is more subject here than there to impairment—a change, as has been before intimated, in which the latter does not participate. The reason of this, I am inclined to believe, is owing more to the difference in the habits and modes of living of the inhabitants of the two countries than that of any difference in the salubrity of the climates.

As teeth that are neither too large nor too small, and that have a close compact structure, and slightly tinged with yellow, are indicative of a constitution, whatever it may be at the present time, that was innately good, so those that are long, narrow, and faintly tinged with blue, as well as those that greatly exceed the ordinary size of these organs, and that are irregular in shape, and that have a rough and muddy appearance, furnish assurance of a constitution, that was at least, originally bad. The first of the latter descriptions of teeth are more frequently met with among females than males, and among those of strumous temperaments, than those in whom this diathesis does not exist. There is another kind of teeth that resemble these in shape and size, and also, in texture, that have been more universally regarded as denoting a tendency to phthisis pulmonalis, than any other description that have attracted the attention of writers on this disease. They are characterized by whiteness, and a pearly gloss of the enamel, and are thought by many to be exceedingly durable, but I have observed that individuals who have this sort of teeth, when attacked by febrile or any other form of malady that had a tendency to alter the fluids of the body, were very subject to tooth ache and dental caries, and that when this condition of the general system was continued for a considerable length of time, that their teeth, in rapid succession, crumbled to pieces.

It would seem, from this circumstance, that the fluids of the mouth, in subjects of strumous temperaments, if free from other maladies, or tendencies to them, are less prejudicial to the teeth than those of persons of most other constitutions, and I am of the opinion that it is owing to this that they are so seldom attacked with caries. M. Delabarre, in connection with a passage from which I have before quoted, in relation to the softening of the interior of the teeth, takes occasion to allude to the effects produced upon these organs, in those who accidentally become consumptive. He is of the opinion that caries supervenes to the disease, and is a consequence of the general debility that is engendered by it. He says, however, that "the patient generally dies before the central ganglion arrives at that state in which its properties are changed."*

Now, this is directly opposed to all observation on the subject, for it is well known that teeth are less affected by this disease than almost any other, and it is unfortunate for the doctrine which he has espoused (that the bony tissue of these organs are softened by the ceasing of the arteries to supply it with calcareous materials) that he should have resorted to this argument. Its absurdity is rendered apparent by his own showing, and that, too, in the paragraph succeeding to the one in which it is used. He says, "whatever may be the diseased condition of the teeth, they may be examined as unexceptionable evidence, that will inform us whether the patient owes his present state of health to a predisposition, or whether having supervened during the course of his life, it depends on an accidental cause."

If the state of the health, subsequent to the ossification of the teeth, were capable of diminishing or increasing the density of these organs, we could learn nothing from an inspection of them concerning the primordial constitution. Nor would we, therefore, be able to determine whether the present state of health was the result of constitutional predisposition, or that of some other cause; for, if they were subject to changes, like other parts of the body, their physical condition might be different to-day from what it was yesterday, so that a diagnosis, founded upon the character of the teeth, would be nothing more than mere vague conjecture.

But, although Delabarre is, in many things, somewhat inconsistent, very many of his views are correct, and few men have

* Vide, Traite, de la Seconde Dentition, page 265.

contributed more largely by observation and experience, to the advancement of the science of the teeth than he has done.

In speaking of persons who have that kind of teeth, which, though beautiful, from having smooth and apparently polished surfaces, present shades intermixed with a dirty white, he says, they "have had alternations of good and indifferent health during the formation of the enamel." "These teeth," he continues, "ordinarily have elongated crowns, and may present marks of congenital atrophy." Again he observes, "teeth of this sort deceive us by appearing more solid than they are; they remain sound until about the age of fourteen or eighteen; then, at this period, a certain number of them decay, especially when in infancy, the subject was lymphatic, and continues to be so in adolescence. This description of teeth is frequently met with among the rich classes, where children born feeble, reach puberty only by means of great care, and, consequently, owe their existence only to the unremitting attention of their parents, and the strengthening regimen that the physician has caused them constantly to pursue. Having reached the eighteenth or twentieth year, their health is confirmed, but the mucous membranes ever after have a tendency to be affected; the redder colour of the mouth, more especially its interior, and that of the lips, and the upper part of the palate; which, by degrees, discovers itself as the subject gradually advances in existence, shows its ameliorated condition. It is thus, that numerous persons, having gained a sanguinous temperament, would deceive us, if it were not that some marks of erosion are seen on the masticating surfaces of the first permanent molares, which informs us that the present health is the result of amelioration."

There are other cases where the teeth are of so inferior a quality, that they no sooner emerge from the gums than they are seized upon and destroyed by caries, while the subjects who possessed them, as has been intimated in another place, were enabled, by skilful treatment, to overcome the morbid constitutional tendencies, against which, during the earlier years of their existence, they had to contend, and eventually, to acquire excellent health. But in forming a prognosis, it is essential to ascertain whether the general organical derangement that prevented the teeth from being well formed, and thus gave rise to their premature decay, is hereditary, or whether it has been produced by some accidental cause subsequent to birth. The procurement of health in the former case, will be less certain than in the latter, for, when the original elements of the organization are bad, the attainment of a good constitution is by far more difficult.

The denudation of the bony tissue of the teeth by the scaling of the enamel is an affection, which though rare, that is nevertheless occasionally met with. It is called by Duval the "*decorticating*,"* of the teeth, and this designation is by no means inappropriate, as it at once conveys a correct idea of the true character of the disease.

The kind of teeth that seem most liable to this affection are met with in persons of sanguino-mucous temperaments, and that have suffered in early childhood from general febrile or inflammatory disease. From this circumstance it is highly probable that the intermediary substance,† or that which connects the enamel with the bone is either partially destroyed or so impaired during the formation of the teeth, by the constitutional derangement, as to prevent it from constituting as perfect a bond of union between the two as may be necessary to their mutual support and protection. Admitting this hypothesis, the scaling of the enamel,

* See Dictionary of Medical Sciences, art. Dent.

† I speak of an intermediary substance, (one of the offices of which is supposed to be the connection of the enamel with the bony tissue of the tooth,) because its existence has been conclusively demonstrated. The discovery of this, if I mistake not, was first made by Dr. H. H. Hayden, and described in a letter addressed to Dr. Mitchell, and published in the *Medical Repository,* in 1813. He says, "I mentioned to you," (alluding to a conversation which he had previously had with Dr. M. on the subject,) "that I had noticed a substance entering into the composition of the human teeth, and those of animals, situated between the enamel and the bone: and apparently differing in substance from either; that in the case in which I first discovered it, (which was a very large human tooth, that had gone in the most rapid manner to decay;) the appearance of this substance was similar to that of the membrane which lines the shell of an egg." The manner by which he made the discovery of this substance, he describes in another part of the letter. The existence of this substance is also particularly mentioned by Berzelius, and Professor Retzius, but in such a way as leads me to conclude, that they were not aware that Dr. H. was the first who discovered it.

when attacked externally with erosion, is easily accounted for. Its connection with the subjacent bone being weak or imperfect, the destruction of the integrity of its fibres cause it to flake off from the teeth. Thus, the malady may be regarded as the result of an imperfect union of the enamel with the bone, and the action of chemical agents upon its external surface.

The teeth of persons who have suffered from small-pox, measles, or other severe forms of eruptive disease or malignant inflammatory fever, during the time of their enamelling, often present a very singular appearance, unlike those that are affected with the ordinary kind of erosion, where the edges of the enamel around the part affected are rough and brittle, they are smooth, or comparatively so, while the injury is confined to only very small and regularly circumscribed spots which extend in a direct horizontal line across such of the teeth as have been attacked by it. These pits are sometimes so close to each other that half a dozen or more of them are united so as to form a sort of narrow and irregular groove. Two and sometimes more of these grooves or rows of pits extend across the same teeth, but this happens only in those cases, in which the subjects have either had relapses, or been affected, during the enamelling process, with more than one of the maladies that give rise to their formation.

There are many other characteristics which the teeth present in shape, size, density and colour, and from which, valuable inductions might be made, both with regard to the innate constitution and the means necessary to their own preservation; but as the limits I have prescribed to this inquiry will not admit of their consideration, I shall conclude this part of my subject, by observing, that the appearances of these organs, as I have before said, vary almost to infinity. Each is indicative of the state of the general health at the time of their formation,—of their own physical condition and susceptibility to injury.

### OF THE PHYSICAL CHARACTERISTICS OF THE GUMS.

Little can be ascertained concerning the primordial constitution from an inspection of the gums. Subject to the laws of the general economy, their appearance varies with the state of the constitutional health and the condition and arrangement of the teeth. Although the immediate or proximate cause of disease in them may be regarded as local irritation,—produced by depositions of tartar upon the teeth, or decayed, dead, loose, or irregularly arranged teeth, or a vitiated state of the fluids of the mouth, resulting from general organical derangement, or any or all of the first mentioned causes, their susceptibility to deleterious impressions, is influenced to a very considerable extent, by the constitution; and, the state of this, determines too, the character of the effects that are produced upon them by local irritants. For example, the deposition of a small quantity of tartar upon the teeth, or a dead or a loose tooth, would not, in a healthy person, of a good constitution, give rise to any thing more than a slight redness or tumefaction of the margin of the gums in immediate contact with it, while in a scorbutic subject, it would cause it to assume a dark purple appearance for a considerable distance around, to become flabby, more turgid, and to separate and retire from the necks of the teeth, or to grow down upon their crowns, to ulcerate and bleed from the slightest injury and to exhale a fœtid odour. And, in proportion as this disposition of body exists, their liability to be thus affected is increased; and, it is only among constitutions of this kind, that that peculiar preternatural prurient growth in them, by which the whole of the crowns of the teeth sometimes become almost entirely imbedded in their substance, takes place.

But notwithstanding the dependency of the condition of the gums upon the state of the constitution, they are occasionally affected with sponginess and inflammation in subjects of the best temperaments and of uninterrupted good health. The wrong position of a tooth, by causing a continued tension of that part of the gum which invests its alveolus, sooner or later, gives rise to a sort of chronic inflammation in it, and the alveolo-dental periostea, and a gradual wasting of its substance, about the mal-placed organ. Tooth-ache too, from whatever cause it originates, often produces the same effects, and the formation of salivary calculus upon the teeth, however small the quantity, is likewise prejudicial to their health.

All of these may occur independently of the state of the general health. A bad arrangement of the best constituted teeth, and tooth-ache, may be produced by a multitude of accidental causes which have no connection with the operations of any of the other parts of the body.

Therefore, while the appearance and physical condition of this peculiar and highly vascular structure, are influenced, in no inconsiderable degree, by the habit of body, they are not diagnostics that always and with unerring certainty indicate the pathognomic state of the general system. It can, however, in by far the larger number of cases, where the gums are in an unhealthy condition, be readily ascertained, whether the disease is altogether the result of local irritation, or whether it has been favored by a constitutional tendency. A comparison of the different effects, that are produced upon them by the same causes, in different individuals, will enable a careful observer, in most instances, to decide without much difficulty.

In adverting to the influence of local causes in giving rise to disease in the gums, Mr. Thomas Bell,* says, "But, although it appears that local causes are most frequently the agents in producing diseased action in these parts," (the gums and alveolar processes,) "yet we find occasionally that similar affections are proved to arise from constitutional sources alone, by their attacking parts of the gum where no irritating local cause remains, or by their continuing unabated, after the removal of such teeth as might be supposed to have produced them."

The agency of an altered condition of the juices of the mouth in the production of disease in the gums is too much overlooked. The irritation produced by the salivary and mucous fluids of this cavity is often as great as that which arises from a decayed, dead or aching tooth, or a deposite of tartar upon the teeth, and I have not the least doubt that they are the exciting and immediate causes of many of the maladies that attack these parts, and which are supposed to result alone, from constitutional predisposition. The effects which they produce upon the teeth when in a morbid state has already been shown. It is well known too, that they readily oxidize silver, and sometimes tarnish eighteen and even

* See Anat. Phys. and Diseases of the Teeth, page 207.

twenty carat gold. That they are irritating, when in this condition, to the soft parts of the mouth, is rendered still more conclusive from the fact that when allowed to escape and come in contact with the cutis of the lips or chin, which is continuous with the mucous membrane of this, as well as that of all the other natural cavities of the body, they not only increase its sensibility, but also not unfrequently produce painful excoriations.

Although the inference drawn by Mr. Bell, is in accordance with the pathognomic doctrines of the present day; I do not think it warranted by the premise, for, as I have shown, there may be other agents in the mouth than the teeth, capable of producing irritation.

I, however, fully concur with him in the following opinion: "The occurrence," says he, "of malignant ulcers, apparently attacking first of all, the gums and alveolar processes, and spreading from thence to the body of the jaw-bone, the cheek, the antrum, the orbit, &c., is too frequent not to have attracted the attention of every surgeon who has seen much of disease. Whether the irritation produced by the teeth, or whether any other source of local irritation, be the exciting cause, it is to the constitution that we must look for that predisposition, without which these severe and often fatal affections could not occur; and although, in some cases, the removal of the local irritant, in the very early stage of the complaint, may probably arrest its progress for a time, there is seldom much advantage attending it after the neighbouring parts have once become the seat of such disease."

Disease in the gums or alveolar processes, or both, I am of opinion, would seldom occur, no matter what the constitutional predisposition be, were it not for local exciting agents of some kind or other; nor, would it, on the other hand, ever assume a malignant form or character, were it not for some general tendency to such malady. And, when once implicated in disease of a malignant kind, the parts, though the exciting cause be removed, are seldom, as Mr. Bell observes, able to recover their healthy tone.

Both medical and dental practitioners can testify to the truth of this, and to the latter, in the treatment of the more aggravated forms of spongy and inflamed gums, or as the affection is sometimes called, "conjoined suppuration," it has been but too frequently verified. Although the progress of the malady may, for a time, be arrested, it is almost certain, sooner or later, unless the most thorough preventive means be constantly employed, to return; and, in the preventing of which, the difficulty is always in proportion to the depraved condition of the general system.

Influenced then as the diseases of the gums are by the state of the constitutional health, it is necessary in the treatment of their diseases, to discriminate between those that have resulted from local irritation alone, and those, which, though this was their immediate and exciting cause, are nevertheless aggravated by some peculiar physical depravity.

It is a well-established axiom in pathology, that each disposition has its own peculiar tendencies, and that these tendencies are increased by an augmentation, from whatever cause produced, of the general irritability of the system. Thus, in a person of a mucous disposition, a derangement of the digestive organs aggravates the habit, and increases the tendencies superinduced by it, to certain forms of disease in particular organs, and in none more than the gums. For example—a local irritant that would cause in an individual of this disposition, and not affected with any appreciable general derangement, nothing more than a slight congestion in the margins and apices of the gums between the teeth, would occasion in another of the same disposition, if affected with dyspepsia, turgidity and sponginess of their substance for a considerable distance around.

Enfeeblement of the vital powers of the body, increases the susceptibility of the gums to the action of irritating agents. Hence, they are often affected with inflammation, sponginess and suppuration of their edges, in persons labouring under excessive grief, melancholy, or any other affection of the mind, tending to enervate the physical energies of the organization. Disease, therefore, in these organs, is always favoured by general debility, and the reason of its being more aggravated in some than others, is owing to the differences in the attention that is paid by different individuals to the cleanliness of the teeth, and the differences in the tendencies that exists in them to it. In dispositions unfavourable to diseased action in the mucous membranes, and where constant and regular attention to the cleanliness of the teeth is observed, the only notable effects resulting to the gums from debility, is a diminution of their colour.

A local disease, situated in a remote part, often has the effect of diminishing the tendency in the gums to be morbidly excited, but when from its violence or long continuance, the general health becomes implicated to a considerable extent, the susceptibility of these parts is augmented.

Although deriving the predisposition which they have to disease from a specific, morbid, constitutional tendency, they, nevertheless, when diseased, contribute in no small degree to derange the whole organism. An unhealthy action here vitiates the fluids of the mouth, and renders them unfit for the purposes for which they are designed,—hence, when these parts are restored to health, whether by the loss of the teeth, or the prescription of the dental surgeon, the improvement that immediately takes place in its condition.

But, not only is a healthy condition of the gums essential to the general health, but it is likewise essential to the healthy condition of the teeth and their retention in their sockets. From the intimate and direct relationship that subsists between them, disease cannot exist in one, without, in some degree, at least, affecting the others. Caries of the teeth, for example, gives rise to inflammation of the gums, and the alveolo-dental periostea. On the other hand, inflammation in these parts is always, sooner or later, followed by a gradual destruction of their own substance, and that of the alveolar processes, the loosening, and frequently the falling out of the teeth,—a morbid condition of the juices of the mouth, from which, decay of the teeth, when the organs are not sufficiently dense to enable them to resist their corrosive action, and an impairment of the vital powers of the body, is almost certain to result.

But, having offered these few general remarks, I shall proceed to notice some of the more striking of the characteristics of the gums, and their local and constitutional indications—premising that a minute description of their various pathological conditions, as influenced by the different states of the general system, and habits of body, constitutes no part of the design of this inquiry. A full and accurate description of their various phenomena, together with their numerous relationships, would fill a volume of itself. I must, therefore, leave the performance of that task to some other pen, or occasion.

In childhood, or during adolescence, when the formative powers of the body are all in active exercise, and the nervous susceptibilities of every part of the human frame highly acute, the sympathies between the gums and other parts of the organism, and particularly the stomach, are, perhaps, greater than at any other period of life. The general health, too, at this time, is more fluctuating, and with all the changes this undergoes, the gums vary. Moreover, there are operations which are carried on beneath and within their substance, which are almost constantly altering their appearances and physical characteristics,—and which, being additionally influenced by various states of health and dispositions of body, it may readily be conceived, that those which are met with in one case, might be looked for in vain in another.

Having arrived at that age when all the organs of the body are in the full vigour of maturity, and not under the debilitating influences to which they had been subject during the earlier periods of life, the gums participate in the happy change, and as a consequence, present less variety in their characteristics. The general irritability of the system is not now so great, the gums are less susceptible to the action of irritating agents, and as a consequence, less frequently affected with disease; but as age advances, and the vital energies begin to diminish, the latent tendencies of the body are awakened, and they are again easily excited to morbid action.

In subjects of the most perfect constitutions, and during adolescence, they present the following appearances. They have a violet colour, a firm consistence, rough surface, their margins form along the outer surfaces of the dental circle, beautiful and regular festoons, and their mucous membrane, as well as that which covers all the other parts of the mouth, have a fresh, lively, roseate appearance.

The time for the molting of a primary tooth is announced some weeks before it takes place by an increased redness and slight tumefaction of the edges and apices of the gums surrounding it. The dentition of a tooth, whether of the first or second set, is also preceded by similar phenomena in the gum through which it is forcing its way, and these will be the more marked as the condition of the system is unhealthy, or as the habit of body is bad.

If the health of the subject continues good, and the teeth be well arranged, and their crowns do not wear off, and the necessary attention to their cleanliness be strictly observed, the characteristics just enumerated will be preserved through life, except that there will be a slight diminution of colour in them, from after the age of puberty until that of the next climacteric period of life, when they will again assume a somewhat redder appearance. But if the health of the subject becomes impaired, or the teeth be not regularly arranged, or wear off, or be not kept free from all lodgments of extraneous matter, the edges of the gums, and particularly their apices between the teeth, will inflame, swell, and become more than ordinarily sensitive.

The gradual wasting or destruction of the margins of the gums around the necks of the teeth which sometimes takes place in persons of the best constitutions, and is supposed by some writers to be the result of a general atrophy, is ascribable, I have not the least doubt, to some one or other of these causes,—favoured, perhaps, by a diminution of vitality in the teeth, whereby they are rendered more obnoxious to the more sensitive and vascular parts within which their roots are situated. This explanation of the causes of the malady, (for it is evidently the result of diseased action in the gums,) is rendered more than probable, by the fact that, it never occurs with those, who, from early childhood, have been in the regular and constant habit of thoroughly cleansing their teeth from four to five times a day.

Mr. Bell, however, while he thinks it may occasionally be an "indication of a sort of premature old age," does not believe it can "always be thus accounted for, as it is sometimes seen in young persons," and "doubtless arises," he says, "from the same cause as those presently to be considered," (alluding to what he afterwards says upon the same subject,) "as originating a similar loss of substance in these parts, when attended with more or less of diseased action." I cannot, for reasons that have been already assigned, concur with him in opinion that it "occasionally takes place without any obvious local or constitutional morbid action."*

Although possessed, as those, in the larger number of cases are, whose gums are such as I have just described, with the best of constitutions; they may, by intemperance, debauchery, or long privation of the necessary comforts of life, or protracted febrile or other severe kinds of disease, have their assimilative and all their other organs, so enervated, as to render every part of the body highly susceptible to morbid impressions of every sort, but still, this general functional derangement, rarely predisposes the structure now under consideration, to any of the more malignant forms of maladies that are occasionally known to attack it in subjects possessed of less favourable innate constitutions. Its margins may inflame, become turgid, ulcerate, and recede from the necks of the teeth, and the whole of its substance be involved in disease, but it will seldom be attacked with scirrhus or fungus tumours, or bad conditioned ulcers, or affected with morbid preternatural prurient growths; and in the treatment of its maladies, we can always form a more favourable prognosis in persons of this description, than those coming into the world with some specific morbid constitutional tendency.

But, the occurrence of severe constitutional disease even in these subjects, is followed by an increased irritability of the gums, so that the slightest cause of local irritation, gives rise to an afflux of blood to, and stasis of this fluid in their capillaries.

The teeth of persons thus happily constituted, are endowed with characteristics, such as have been represented as belonging to those of the best quality. They are of a medium size, both in length and volume, of a dull or heavy white, compact in their structure, generally well arranged, and seldom affected with caries.

Pursuing the subject, another constitution is observed, in which the gums, though partaking somewhat of the characteristics of those just described, yet differ from them in some particulars. Their colour is of a deeper vermilion; their edges rather thicker, their structure less firm, and their surface is not so rough, but

* Vide Anat. Phys. and diseases of the teeth, p. 210.

more humid. Their mucous membrane has a more lively and animated appearance. They are rather more sensitive and susceptible to the action of local irritants, and their tendencies are more increased by general organic derangement, than they are in gums possessed of the appearances first mentioned.

Their diseases, though generally easily cured or arrested by proper remedial treatment, are nevertheless more obstinate, and when favoured by disease of the general system, assume a still more aggravated form. Their predisposition in fact, to disease, is so much increased by severe and long continued general morbid excitement, and especially during youth, and by febrile or inflammatory affections, or obstructions in the parenchymatous organs, that not only their margins, but their whole substance also, sometimes becomes involved in inflammation, and sponginess, followed by ulceration of their edges, and a recession of them from the necks of the teeth, which, in consequence, loosen, and often drop out. But gums of this kind, like those first described, seldom grow down upon the crowns of the teeth. Neither are they often attacked with scirrhus or fungus tumors, or any form of malady resulting in sanious or other malignant conditioned ulcers. With diseases of this kind, they are not perhaps ever affected, except in those cases where every part of the body has become exceedingly depraved; and this is an occurrence that happens much less frequently in habits originally good, than in those in whom a specific tendency to such unfavourable morbid constitutional diathesis was primitively implanted in the organization.

The teeth of those whose gums are possessed of this second description of characteristics, if well arranged, and kept constantly clean—and, if the secretions of the mouth be not vitiated by general disease, will, in most cases maintain their integrity through life.

It is only among sanguinous persons that this description of gums is met with, and the teeth of subjects of this kind are generally of an excellent quality, and though rather more liable to be attacked with caries than those first noticed, it is seldom that they are affected with it.

In sanguino-serous and strumous dispositions, the gums are paler than in either of the preceding, and though their margins are thin and well festooned, they exude, after the twenty-fifth or thirtieth year, a small quantity of muco-purulent matter, which on pressure, sometimes is seen to ooze from between them and the necks of the teeth. Their texture is usually firm, and they are not very liable to become spongy, and they often remain in this condition to a late period of life without undergoing any very perceptible change. Although their connection with the necks of the teeth and alveolar processes appears weak, they rarely separate from them.

In remarking upon these kinds of dispositions, M. Delabarre tells us, that if they "abuse their physical powers," by an injudicious regimen, or too much study, they become enervated and "are subject to chronic sanguinous obstructions of the capillaries of the lungs, and to profuse hemorrhages." Dyspepsia and diseases in which the primæ viæ generally, is more or less involved, and chronic hepatitis are not unfrequent among subjects of this kind, and are indicated by increased irritability, and sometimes a pale yellowish appearance of the gums. In jaundice, the yellow serosity of the blood is very apparent in the capillaries of this structure.

These constitutions are more common to females than males, to the rich than the poor, and to those of sedentary habits than to those who use invigorating exercises. If at any time during life the health is ameliorated, the gums assume a fresher and redder appearance, and the exudation of muco-purulent matter from between their edges and the necks of the teeth ceases.

In mucous dispositions, the gums have a smooth, shining appearance, and are rather more highly coloured than those of the preceding. Their margins, also, are thicker, more flabby, and not so deeply festooned; they are more irritable, and, consequently, more susceptible to morbid impressions.

If to this disposition, there be combined a scorbutic or scrofulous tendency, the gums during early childhood, in subjects, which, from scanty and unwholesome diet, have become greatly debilitated, are liable, besides the ordinary forms of disease that attack them, to another,—characterized by their separation from, and exfoliation of the alveolar processes—accompanied by a constant discharge of sanies. This malady, however, though peculiar to childhood, and wholly confined to the indigent, is by no means common.*

These constitutions are rarely met with, except in low, damp, and sickly districts of country. Their mucous membranes are exceedingly irritable, and secrete a large quantity of fluid.

In alluding to this species of disposition. M. Delabarre says, "in children, the skin is ordinarily white and tender; nevertheless, it is sometimes brown and wrinkled. They are usually fragile and weak; their blood is pale, their nutrition is imperfectly effected. In females, the vertebrial column is disposed to curve about the age of puberty, because," says he, "at this period, the vital energies are principally directed towards the uterus, and in consequence, although so very necessary in the osseous system, there they appear to be weak.

"The number of observations that I have collected during my practice in the city, and in several public institutions, have confirmed me in the opinion, that it is in this constitution, especially (alluding to the mucous) that the children of whom I have just spoken, are met with. The *organic life* in them has so little energy, that the operation of a local cause on a certain point, with greater activity than it has before done, sensibly diminishes the assimilative force of almost all the others. It is also probable, that the development of ganglion obstructions during dentition, are, many times, owing to the diminution of the sensibility in the lymphatics.

"We may also remark," says he, "that, their skin being very susceptible, the sympathy established between it and the mucous membranes, renders individuals of this kind very liable to contract rheums, and gastric and intestinal affections; they are likewise subject to easy night sweats, and vomitings of a sero-mucous fluid,"† &c.

But, persons even thus unhappily constituted, do, sometimes, by a change of residence and judicious regimen, acquire tolerably good constitutions. Little advantage, however, is derived from these, unless they are had recourse to before the twenty-fifth or

* See an article from the author on this malady, in the Maryland Medical and Surgical Journal, vol. 1, page 445.

† Vide, Traite de la seconde dentition, pagé 287–8.

thirtieth year of age, though they may prove beneficial at a much later period.

The gums, in subjects in whom there exists a scorbutic tendency, have a reddish-brown colour; their margins are imperfectly festooned, and thick; their structure rather disposed to sponginess, and ever ready on the presence of the slightest cause of local irritation, to take on a morbid action. When thus excited, the blood accumulates in their vessels,—where, from its highly carbonized state, it gives to the gums a dark, purple, or brown appearance; they swell, and become spongy and flabby, and bleed from the slightest touch. And to these symptoms, supervene the exhalation of a fœtid odour, the destruction of the bond of union between them and the necks of the teeth, suppuration and recession of their margins from the same,—gradual wasting of the alveolar cavities, the loosening and not unfrequently the entire loss of several or the whole of the teeth. These are the most common results, but sometimes they take on other and more aggravated forms of diseased action. Preternatural prurient growths of their substance, fungus and scirrhus tumours, ichorous and other malignant conditioned ulcers are occasionally met with here, in persons in whom there exists a scorbutic taint.

The occurrence of aveolar abscess in dispositions of this kind is often followed by necrosis and exfoliation of portions of the maxillary bone, and the effects which result to the gums from it are always more pernicious than in habits less depraved.

The development of the morbid changes that take place in this structure, even in subjects of this kind, while their character is influenced, if not determined, by a specific constitutional tendency, are nevertheless referable to an immediate or proximate cause, and, were this the proper place, I could cite numerous cases tending directly to the establishment of this pathological position, but as this constitutes no part of the design of this inquiry, I shall content myself with what I have already said.

In scrofulous dispositions the gums have a pale bluish appearance, and when subjected to local irritation, they become flabby, exhale a nauseating odour, detach themselves from the necks of the teeth, and their apices grow down between the teeth. The blood circulates through them languidly, and debility seems to pervade their whole substance. They are exceedingly irritable, and not unfrequently take on a very aggravated form of disease, and, as it often happens, that to this, as well as the preceding habit, there are combined tendencies which favour the production of ill-conditioned tumours and ulcers, these are often here met with.

The indications furnished by the gums of a mercurial diathesis in the system, are morbid sensibility, increased vascular and glandular action, foulness, bleeding from the most trifling injuries, pale bluish appearance of their substance, turgescence of the points or apices between the teeth, and sloughing. The effects, however, resulting to these parts from the use of this medicine differ in different individuals according to the general constitutional susceptibility, the quantity taken into the system, and the length of time its use is continued. In persons of very irritable habits, a single dose will sometimes produce ptyalism, and so increase the susceptibility of the gums, that the secretions of the mouth, in their altered state, will at once rouse up a morbid action in them.

The influence of a mercurial diathesis upon these parts, is not unfrequently so great as to result in the loss of the whole of the teeth; but with these effects both the dental and medical practitioner are too familiar to require any further description from me.

Finally, I would observe, that the indications of the several characteristics to which I have now briefly alluded, may not be correct in every particular, and there are others which I have not mentioned; yet, that they will generally be found so, I am persuaded every one whose attention has been for any considerable length of time particularly directed to the subject, will agree. As a general rule, persons of a full habit, though possessed of mixed temperaments, and in the enjoyment of what is usually called good health, have gums that are well coloured, with rather thick margins, and that are very susceptible to local irritation, and with this description of individuals, inflammation, sponginess and suppuration of the gums are very common. To prevent this, constant attention to the cleanliness of the teeth is indispensable.

Professor Schill, says the "gum is pale in clorosis and anæmia; of a purple red colour before an active hemorrhoidal discharge, and in cases of dysmenorrhœa; of a dark red colour, spongy, and bleeding readily in scurvy and diabetes mellitus, and after the use of mercury. Spongy growths indicate caries of the subjacent bone."*

Regular periodical bleedings of the gums in dysmenorrhœa, and particularly in scorbutic and mucous subjects, are not unfrequent, nor in any case where they are in a turgid and spongy condition. Dr. Hayden communicated a very interesting case of this kind to me about two years since, that had come under his own immediate observation.

Spongy growths of the gums in scorbutic and scrofulous persons, often result from irritation produced by decayed teeth, and are not, therefore, always to be regarded as an indication of caries of the subjacent bone.

In treating upon inflammation, sponginess and suppuration of the gums, Dr. Hayden mentions, "great exercise of mind, melancholy, bad diet, suppression of the hemorrhoids, the sudden closing up or healing of seatons, issues," &c. and the "repercussion of some prevailing cutaneous diseases," &c. as favouring the production of this disease.†

The same author also says, he has "observed that persons subject to gout or rheumatism, though corpulent, are seldom affected with this disease," (conjoined suppuration.) "On the contrary," he observes, "I have remarked that persons afflicted with suppuration of the gums, are seldom troubled with gout or rheumatism; if so, however, the paroxysms are slight and of short duration."

Mr. George Waite says, "a change of residence to a damp climate will often rouse up in the gums a great degree of vascularity. In the damp places of England and Ireland, the appearances which the gums present are of a turgid and vascular nature. In the damp countries of France these conditions of the gums run a much greater length from the circumstance of the difference in the constitutions of the two nations. In the damps of Germany and Switzerland, persons also lose their teeth early in life, the

* Outlines of Pathological Semeiology, page 168 of the Select Medical Library edition.

† Vide Med. Recorder for 1822, vol. v, p. 24.

climate engenders malaria and low fevers, enfeebles the powers of digestion, and brings on rheumatic affections with languor and general constitutional debility."

Of the correctness of Mr. Waite's observations there can be no question, and they go to establish what I have said in regard to the predisposing cause of disease in the gums,—namely, that the enervation of the vital powers of the body, from whatever cause produced, increases their susceptibility to morbid impressions.

### OF THE CHARACTERISTICS OF SALIVARY CALCULUS.

The colour, consistence, and quantity of salivary calculus, or tartar, as it is most commonly called, varies in different temperaments, and upon all of which, the state of the general health exercises a considerable influence. They, therefore, furnish diagnostics, important both to the physician and dental surgeon. The indications of the characteristics of this substance are in many cases less equivocal than those of the appearances of any other part of the mouth; but, like those of the gums, should not, perhaps, be alone always relied upon. In the forming of a prognosis, we should not neglect to interrogate every part from which information can be derived concerning the pathognomic condition of the several organs of the body.

Salivary calculus is composed of earthy salts and animal matter. Phosphate of lime and fibrina, or cartilage, are its principal ingredients; a small quantity of animal fat however, enters into its composition. The relative proportions of its constituents vary according as it is hard or soft, or as the temperament of the individual from whose mouth it is taken, is favourable or unfavourable to health; and hence it is, as M. Delabarre remarks,* that the analysisis that have been made of it by different chemists differ. No two give the same results.

The black, dry tartar, says the author to whom I have just referred, which is deposited around the necks of the teeth of such only as are possessed of good constitutions, is never in large quantity, is dissolved in muriatic acid with difficulty, while the dry, yel-

* See chapter on Salivary Calculus in Traite de la Seconde Dentition.

low tartar found upon the teeth of bilious persons dissolves more readily in it; but the soft white tartar, found upon the teeth of individuals of mucous temperaments, he tells us, "is scarcely at all soluble in the acids," but, "is readily dissolved in the alkalies."

In regard to the sources from whence this substance proceeds, there is considerable variety of opinion. English and American writers on the subject agree in ascribing its production to the saliva, but the French have supposed it to proceed from different sources. *Jourdain*, thinks it is secreted by glands, which he believes are scattered over the periostea of the teeth. *Gariot*, says, it comes from the gums. *Serres*, announces to the world that he has discovered upon the mucous membrane of the gums certain glands, whose particular function it is, to secrete this substance. In commenting upon the views of this last mentioned author, M. Delabarre, thus remarks: "The small glands, which he thus designates," (alluding to the appellation of dental which the author gives them,") "may perhaps belong to the mucous or salivary systems, for the saliva, as all physiologists know, is not alone furnished by the parotid glands, but by a great number of calculus kennels, that are very observable in ruminating animals, scattered over various parts of the mucous membrane of the mouth. I, therefore, am of opinion, that this is a gratuitous supposition on the part of this author, because children of a very early age are not affected with tartar, and it is on them that he believes he has discovered the glands, which produce it. Did these really exist, they would augment in size, instead of decreasing, as age advanced, and their functions becoming more and more established, they would attain to a very large size in old persons, and those most subject to tartar. Now, there is nothing to lead us to suppose their existence in these individuals. Therefore, to suppose that organs that have no functions may be very perceptible, which, when they have them, cannot be discovered, is contrary to sound philosophy; were we to do so in this case, the *dental glands* of the author, would be entirely different from all others, which are the more decided, the more they are in action. Inadmissible then, as this supposition is, I do not believe in the existence of these glands, which I have patiently searched for, but in vain."

My own views in relation to the supposed existence of the dental glands of M. Serres, are so fully expressed in the foregoing remarks of M. Delabarre, that I do not deem it necessary to say any thing more upon the subject, except to add, what has been suggested by others, that he has evidently mistaken the mucous follicles of the mucous membrane of the gums for glands.

But, M. Delabarre is not more fortunate in the theory which he advances, of the formation of salivary calculus. He ascribes the production of this substance to a diseased exhalation of the mucous membrane of the mouth. Alluding to what M. Dupuy, professor of the veterinary establishment at Alfont, says concerning the formation of tubercular matter, of a calcareous nature, in soft tissues, where, he supposes there is no other fluid but mucous, he tells us that it is "in the same manner that the exhalents of the gums furnish tartar," and that "they give out more or less of it according as the gums are in a healthy or inflamed state." When diseased, he says, "they are covered with a whitish layer, which is at first soft," but gradually collecting upon the teeth, it afterwards becomes hard; and according to this author, it is only when the gums are inflamed that it is produced.

It is in this way that he accounts for its accumulation on the teeth of one side of the mouth, while those of the other have none of it on them, though they are all bathed alike in the saliva. The concretions of these terreous salts in the salivary conduits, he accounts for, by supposing them to be furnished by the exhalents of the mucous membrane which lines them, and not by the fluid they convey to the mouth.

Analagous formations in other parts he accounts for in the same way. The calculus incrustations that are found, upon a sound, that has remained in the bladder for a long time, on its removal, and from subjects, in whom no previous disposition to gravel had existed, he supposes to be the result of the irritation produced by the instrument in the mucous membrane of this viscus. In replying to this part of his argument in support of his theory that salivary calculus is furnished by the exhalents of the mucous membrane of the mouth, Mr. Bell, says, "The previous non-existence of calculus in the bladder cannot be deemed any proof that the elements of its composition had not been held in solution in the urine, requiring only the occurrence of any extraneous body in the bladder to serve as a nucleus for its deposition. This view of the subject is amply confirmed by the fact, that depositions, both of the lithic salts and of the triple phosphate, the bases of the usual varieties of urinary calculi, are constantly formed from the urine after its expulsion from the bladder."*

It is unfortunate for M. Delabarre, that he drew this analogy, for Mr. Bell has shown it to be conclusive against the theory which he intended to establish by it, "and," says this author, "that salivary calculus, or tartar of the mouth, is deposited in a similar manner from the saliva, is, I think, directly proved, or at least, supported with the highest degree of probability by every circumstance connected with its formation." The fact too, that it is always found in largest quantity on the teeth opposite the mouths of the salivary ducts, is a strong argument of itself, in favour of the theory that it is a salivary production; but, still more conclusive is, that of its formation within the very channels themselves of these conduits.

The theory of M. Delabarre is insufficient for the explanation of its deposition here, for, it is not at all presumable, that an inflammation would seize upon a single point of the mucous membrane of one of these passages, without affecting it to a considerable extent. The most probable cause of its formation in these conduits, therefore, appears to me, to be the accidental precipitation of a particle of it from the saliva on its passage through them, and which, becoming entangled in the mucus, is detained where it afterwards serves as a nucleus for its deposition.

Of the existence of the elements of its composition in the saliva there can be no question. Chemical analysis of this fluid, direct from the glands that secrete it, place all doubt upon the subject at rest. It may also exist in solution in the mucous fluids, but that it does, we are not so well assured.

The circumstance that the deposition of this substance on the teeth is always accompanied by inflammation of the gums, M. Delabarre seems to rely upon as conclusive in favour of the correctness of his views of the manner of its formation. But here

* Anat. Phys. and Diseases of the Teeth, page, 195.

again, he is not more fortunate. The inflammation of which he speaks, is the effect, and not the cause, as he supposes, of its deposition. The soft white layer of tartar, of which he speaks as observable on the gums, when diseased, is nothing more than thick, hardened mucus. I have repeatedly examined it, and am therefore, well assured of the correctness of this assertion.

The deposition of tartar on the teeth of one side of the mouth, without a similar deposite on the corresponding ones of the opposite side, does not furnish the least shadow of evidence in support of the doctrine that this substance is an exhalation from the sanguinous capillaries of the mucous membrane of the gums. The mastication of food, with most persons, is principally performed by the teeth on one side of the mouth, and, with the fact that this function prevents, in a considerable degree, the accumulation of tartar on the organs immediately concerned, every dental practitioner must be conversant. Hence, its frequent collection on those of one side, and not on those of the other. And, that it is ascribable to this, is susceptible of positive proof. If, on the removal of the tartar from the teeth of a person, in whose mouth it had only collected on those of one side, mastication be afterwards altogether performed on these, it will not re-accumulate on them, and if the requisite attention to the cleanliness of his denture be not properly observed, it will soon collect on those of the other side, where it had not before been deposited, or, if deposited, had not remained. I have frequently requested patients to do this, and the foregoing has almost invariably been the result, which, was its formation dependent on inflammation of the gums, would not have been the case.

Again, it often happens that disease is excited in the gums, and of a severe character, by the use of mercurials and other causes, and yet, but a small quantity of tartar collects on the teeth; but, that any condition of the general system, or of the mouth, tending to increase the viscosity of the fluids of this cavity, promotes its formation, is undeniable. There are, however, some temperaments much more favourable to its production than others, and, it is a fact, equally well established, that the mucous membranes of those, in whose mouths it accumulates in largest quantity, are most irritable, and their buccal fluids most viscid.

From all the light, therefore, that has been thrown upon this subject, the conclusion, that this earthy matter is a salivary production, to me appears irresistible, and, the following seems to be the manner of its formation.

It is precipitated from the saliva, as this fluid enters the mouth, on the surfaces of the teeth, opposite the openings into the ducts, from which it is poured. To these, its particles become aglutinated by a mucus, that is always found, in greater or less quantity, upon them. Particle after particle is afterwards deposited, until it sometimes accumulates in such quantities that nearly all the organs are almost entirely enveloped in it. It is always, however, found in greatest abundance on the outer surfaces of the superior molares, and the inner surfaces of the inferior incisors, and it is opposite to these that the mouths of the salivary ducts open.

All persons are subject to salivary calculus, but not alike; it collects on the teeth of some in much larger quantities than it does on those of others, and its chemical and physical characteristics are exceedingly variable. It is, sometimes, almost wholly composed of calcareous ingredients; at other times these constitute but about, or little more than one-half of its substance,—the balance being made up of animal matter. Nor is its colour more uniform. Sometimes it is black, at other times it is of a dark, pale, or yellow brown, and in some instances it is nearly white. There is, also, a difference in its density. In the mouths of some persons it has a solidity of texture nearly equal to that of the teeth themselves, while in those of others it is so soft that it can, with ease, be scraped from the teeth with the thumb or finger nail. The black kind is the hardest, and as it approaches in colour the white, it becomes softer.

The effects that result from the presence of this substance upon the teeth are always pernicious, though sometimes more so than others. An altered condition of the fluids of the mouth, diseased gums, and not unfrequently the gradual destruction of the alveolar processes, and the loosening and loss of the teeth, are among the consequences that arise from it. But besides these, other effects are sometimes produced by it, among which the following may be enumerated: tumours, and spongy excrescences of various kinds of the gums; necrosis and exfoliation of the alveolar processes, and portions of the maxillary bones, hemorrhages of the gums, anorexia and derangement of the whole digestive apparatus; foul breath, catarrh, cough, diarrhœa, diseases of various kinds in the maxillary antra and nose, pain in the ear, headache, melancholy, hypochondriasis, &c. The character of the effects, however, both local and constitutional, that are produced by it, depends upon the quantity and consistence of the tartar, and the temperament and state of the general health; and the two former of these, are determined by the two latter, and the attention that is paid to the cleanliness of the teeth. If this last be properly attended to, salivary calculus, no matter how great the constitutional tendency to its production may be, will not collect upon the teeth. The importance, therefore, of its constant observance, cannot be too strongly impressed upon the mind, and especially of those in whom there exists a great tendency to its deposition.

Salivary calculus collects in but very small quantities on the teeth of persons possessed of the most perfect constitutions, and, even on these it is seldom found, except on the inner surfaces of the lower incisors next the gums. It is then black, or of a dark brown, dry, and almost as hard as the teeth, to which it adheres with great tenacity.

It rarely happens that any unpleasant effects arise from the presence of this kind of tartar upon the teeth. The general health is never affected by it, and the only local injury that results from it is a slight turgescence of the edge of the gums in immediate contact with it.

The indications, therefore, of this description of tartar are favourable, both with regard to the teeth, gums and organism generally. The teeth upon which it is found are of an excellent quality and rarely affected with caries. They are possessed of those characteristics which have been represented as belonging to those of the best kind, and teeth of this description are only found among persons of good innate constitutions.

There is another kind of black tartar differing from that which has just been described in many particulars. It is found in the mouths of those, who, though their innate constitutions were good, have nevertheless had their physical powers much enervated by privation of the necessary comforts of life, or disease, or intemperance and debauchery, and most frequently by the last. It is found in large quantities on the teeth opposite the mouths of the salivary ducts; it is exceedingly hard, and is aglutinated so firmly to the organs incrusted in it, that it is with the greatest difficulty that it can be removed from them; it is very black; has a rough and uneven surface, and is covered with a glairy, viscid and almost insufferably offensive mucus.

The presence of this kind of salivary calculus is attended with very hurtful consequences, not only to the gums, alveolar processes and teeth, but also, to the general health. It causes the gums to inflame, swell, suppurate and recede from the teeth,—the alveoli to waste, and the teeth to loosen, and frequently to drop out. The secretions of the mouth are also vitiated by it, and thereby rendered unfit to be taken into the stomach. Hence, as long as it is permitted to remain on the teeth, neither the skill of the physician, nor the best regulated regimen, though they may afford partial and temporary relief, will fully restore to the system its healthy functions.

As this kind of tartar is seldom, if ever met with, except among persons, who have had excellent constitutions, the teeth on which it is deposited are generally sound, and, in this condition, they are often caused, by the effects which it produces upon the gums and alveolar processes, to loosen and drop out. Whole sets of the best constituted teeth, are in this way frequently destroyed.

The dark brown tartar is not as hard as either of the descriptions of black. It sometimes collects in tolerably large quantities on the lower front teeth, and on the first and second superior molares; it is also often found on all the teeth, though not in as great abundance as on these. It does not adhere to the teeth with as much tenacity as either of the preceding kinds, and can, therefore, be more easily detached from them. It exhales a more fœtid odour than the first, but is less offensive than the second.

The persons most subject to this kind of tartar are of mixed temperaments,—the sanguinous, however, almost always predominating. They may, perhaps, be denominated sanguino-serous and bilious. Their physical organization, though not the strongest and most perfect, may nevertheless, be considered very good. But, being more susceptible to morbid impressions, their general health is less uniform, and more liable to impairment than those possessed of the most perfect constitutions.

The effects arising from accumulations of this description of salivary calculus on the teeth, both local and constitutional, are less hurtful than that last noticed, but like that, it causes the gums to inflame, swell, suppurate, and to retire from, and expose the necks of the teeth, the alveoli to waste, the teeth to loosen and sometimes to drop out. A vitiated state of the juices of the mouth, also results from its presence, or rather from the effects which it produces upon the gums and alveolar processes.

Salivary calculus that is of a pale or yellow, brown-colour, is of a much softer consistence than that which is dark, and is seldom found upon the teeth of persons, except those of bilious temperaments, or those in whom this disposition preponderates. It has a rough, and for the most part, a dry surface; it is found in large quantities on the teeth opposite the mouths of the salivary ducts, and it sometimes happens that every tooth in the mouth is completely incrusted with it. It contains less of the earthy salts and more of the fibrina and animal fat than that of any of the foregoing descriptions, and from the quantity of vitiated mucus in it and adhering to it, has an exceedingly offensive smell. It is somemes, though not always, so soft that it can be crumbled between the thumb and finger.

Inflammation, turgescence and suppuration of the gums, inflammation of the alveolo-dental periostea, the destruction of the sockets and loss of the teeth, and an altered condition of the fluids of the mouth, are among the local effects that arise from the long-continued presence of large collections of this kind of tartar on the teeth. The constitutional effects are not much less pernicious. Indigestion and general derangement of all the assimilative functions, are among the most common. If the deposition be not large, inflammation and sponginess of such parts of the gums as are in immediate contact with it, and fœted breath, are the principal of the unpleasant consequence that are produced by it.

White tartar rarely collects in very large quantities, and though most abundant on the outer surfaces of the first and second superior molares, and the inner surfaces of the lower incisors, it is nevertheless frequently found on all the teeth. Its calcareous ngredients are less abundant than those of any of the preceding descriptions. Fibrina, animal fat, and mucus, constitute considerably more than one-half its substance. It is very soft, seldom exceeding in consistence common cheese curd, and to which in appearance, it bears considerable resemblance. Although it exerts but little mechanical irritation upon the gums, it notwithstanding, from its acrid qualities, keeps up a constant morbid excitement in them. Its effects, however, upon the teeth, are by far more deleterious than any other description of salivary calculus. It corrodes their enamels, and causes rapid decay of the organs. The fluids of the mouth are also vitiated by it.

It is only upon the teeth of persons of mucous dispositions, or those who have suffered from diseases of the mucous membranes, or those in whom these have been more or less involved, that this kind of tartar accumulates.

There is one other kind of tartar that is described by dental writers. It, however, as I have on another occasion said,* is of a dark green colour, and is seen more frequently on the anterior surfaces of the upper teeth occupying the front part of the mouth, than on any of the others. Its resemblance is more that of a stain on the enamel than salivary calculus. Children and young persons are more subject to it than adults, though it is occasionally observed on the teeth of the latter. It is exceedingly acrid, and has the effect of decomposing the enamel; the margins of the gums around the teeth having it on them, are inflamed, and the sanguinous capillaries of their whole substance appear to be distended and more than ordinarily languid.

This kind of discolouration of the enamel is indicative of an irritable condition of the mucous membranes and viscidity of the fluids of the mouth. Sour eructations, vomitings, diarrhœa and dysentery are not unfrequent with those whose teeth are thus affected.

### OF THE CHARACTERISTICS OF THE FLUIDS OF THE MOUTH.

In treating upon the physical characteristics of the fluids of the mouth, it will not be necessary to dwell at much length on the effects produced by them, when in a morbid condition, on

* See my Practical Treatise on Dental Surgery, pp. 224-5.

other parts. Concerning their agency in the production of caries of the teeth, I shall add one or two more remarks.

Doctor Samuel K. Mitchell, in a letter addressed to Thomas Charles Hope, M. D. of Edinburg, dated October 10th, 1796, has proven that an acid is formed in the mouth capable of decomposing the enamel and bony structure of these organs. From his remarks upon the subject, I will quote one or two passages. "From the known disposition of oxygen," says this distinguished writer, "to combine with septon, and form the septic acid, it appeared to me probable, that it would be formed occasionally in the human mouth, from the remains of food adhering to the teeth, sticking between them, and corrupting them. If this was the case, this acid ought to unite with the calcareous earth of the teeth, and form the septite of lime, which might be washed away by the spittle, or probably, in some instances, concrete upon the teeth themselves: and, if formed there, the acid might be expected to corrode the enamel, lay bare the bony part, and bring on caries; or incrust the inside, irritate the gums and occasion soreness and bleedings.

"In order to determine whether these things were so, I procured from a dentist a quantity of the substance called 'the tartar of the teeth," which I supposed might contain some septite of lime, and subjected it to a number of experiments. Having sometime before received complaints from the merchants of Glasgow of the faulty quality of the potash supplied from New York; and having been requested by a member of the Chamber of Commerce to visit with him the stores of the inspectors of those articles in the city of New York, I had collected samples of potash and pearlash of the first qualities, with the view of making experiments upon them.

These salts being in their caustic state, had been placed in separate glasses, to attract water and carbonic acid from the atmosphere; and after standing for several months, the ferruginous and earthy parts having subsided, beautiful crystals of the alkali were formed at the bottom of the liquor. A solution of these crystals was made in water that had been boiled sometime, to extract the air, and precipitate some of its earth; and to this solution of potash was added a parcel of the yellowish, earthy matter, scraped from the teeth, which had been previously reduced to powder in a mortar.

Instantly, on mixing them, bubbles of air were set loose and thickly floated on the surface of the mixture; and by their long continuance without bursting, seemed to indicate a sort of tenacity in the fluid, derived from animal mucilage.

The coarser part of the earthy matter, soon sunk to the bottom; but the finer particles took a longer time to settle down; yet, in a few minutes, even before the liquor had become clear, a piece of clean paper dipped in the solution, and dried before the fire, deflagrated on being burned, and emitted numerous flashes and sparkles, after the manner of saltpetre, while no such lucid or radient appearance was evident on setting fire to paper that had been dried after dipping in a solution of the alkali alone."*

If Dr. Mitchell had repeated this experiment with tartar taken from the teeth of different individuals, and of different degrees of density, he would have found that the quantity of septic (nitrous) acid that it contained, varied, and that its presence is more decided in soft tartar than in that which is hard; he would have discovered also, that the acid was contained in the viscid and vitiated mucus and saliva that penetrated it, and adhered to its surface. This is proven by the fact, that a mixture of these fluids when in a vitiated state, instead of tartar, with the alkaline solution prepared in the manner as described by Dr. M. will furnish precisely the same results. If the salivary and mucous fluids employed in this experiment be taken from around and between the teeth of a person affected with bilious or any severe febrile disease, the result will be more satisfactory.

The manner in which I have procured them, has been the following. I took three or four strans of floss silk, and passed them successively round five or six teeth, just under the free edge of their respective gums, and to absorb all the moisture with them I possibly could, I drew the ends backwards and forwards several times. The silk thus used, I then laid aside, and took several other strans, with which I repeated the operation on several other teeth, and so on, until I had passed them round all that were in

* Vide, American Journal of Dental Science, vol. 1, pages 80—1.

the mouth. Thus charged, these several strans of silk were immersed in an alkaline solution prepared in the manner as described by Dr. Mitchell. Into this mixture a piece of clean, white paper was afterwards dipped, and after it had dried, set on fire. In burning, it emitted bright sparks.

The object in obtaining these fluids from between the teeth and under the edges of the gums, is, because they are, generally, retained here longer than they are in any other part of the mouth, and consequently are less pure. The experiment, if made with saliva, as spittle from the mouth of a person in the enjoyment of any thing like tolerable health, will fail to produce the result here described.

The acidity of these juices when in a vitiated and viscid state, may be tested by a more simple experiment, which consists in moistening a piece of blue paper, died with turnsole, with them. If they be obtained from between the teeth and beneath the edges of the gums, they will, in a short time, turn the paper red.

If, then, these fluids, when in a morbid condition, are possessed of acid qualities, and that they are, is, I think, well established, they must, necessarily, exert a deleterious action on the teeth, and that the decay of these organs is attributable to this, I am fully persuaded.

But to return,—the saliva, in healthy persons, having good constitutions, has a light, frothy appearance, and but very little viscidity. Inflammation of the gums, from whatever cause produced, increases its viscidity, and causes it to be less frothy. In a healthy state, it is inodorous, floats upon and mixes readily with water, but when in a viscid or diseased condition, it sinks and mixes with it with difficulty.

Irritation in the mouth, from diseased gums, aphtheous ulcers, inflammation of its mucous membrane, the introduction of mercury into the system, or the taking of any thing pungent into it, increases the flow of this fluid, and causes it to be more viscid than it is in its natural and healthy state.

In treating on the signs from the saliva, Professor Schill, says, "The sympathetic affection of the stomach in pregnancy is sometimes accompanied by salivation, which in this case mostly takes place after conception, and sometimes continues to the time of delivery. It is also soberved to occur in weakened digestion, in gastric catarrhs, after the use of emetics, in mania, in what are called abdominal obstructions, in hypochondriasis and histeria; salivation occurs during the use of mercury or antimony.

"In confluent small-pox, salivation is a favourable sign. If it cease before the ninth day the prognosis is bad. In lingering intermittents, salivation is sometimes critical; more frequently in these affections it precedes the termination in dropsy.

"Diminution of the salivary secretion, and, in consequence of this, dryness of the mouth, is peculiar to the commencement of acute diseases, as also to the hectic fevers occasioned by affections of the abdominal organs. If the flow of the saliva stop suddenly, there is reason to apprehend an affection of the brain.

"Thick viscid saliva occurs under the same circumstances as the diminution of the salivary secretion, especially in small-pox, typhus, and in hectic fevers. It is thin in ptyalism. In gastric diseases where the liver participates, it becomes yellow or green; by the admixture of blood it may assume a reddish colour; in pregnant or lying-in-women, it is sometimes milky; an icy cold saliva was observed by the author in face-ache.

"Frothy saliva from the mouth is observed in apoplexy, epilepsy, hydrophobia, and in the histerical paroxysm."*

Dr. Bell, editor of the Select Medical Library and Bulletin of Medical Science, in a note to the work from which I have just quoted, says, "acid saliva is regarded by M. Donné, as indicative of gastritis, or deranged digestion. Mr. Laycock," he observes, "on the other hand, infers from numerous experiments on hospital patients, that the saliva may be acid, alkaline, or neutral, when the gastric phenomena are the same. In general, Mr. L. remarked, that it was alkaline in the morning, and acid in the evening."

I have had occasion to observe, that the acid quality of the saliva was more apparent, and more common in lymphatic, mucous and bilious dispositions, than in sanguinous or in sanguino-serous persons, and that weakened or impaired digestion always had a tendency to increase it.

M. Delabarre, says, "When this fluid," (the saliva,) "has re-

* Outlines of Pathological Semeiology; edition of the Select Medical Library, pp. 173–4.

mained in the mouth some moments, it there obtains new properties, according to each individual's constitution and the integrity of the mucous membrane, or some of the parts which it covers.

"In subjects who enjoy the best health, whose stomach and lungs are unimpaired, the saliva appears very scarce, but this is because it passes into the stomach almost as soon as it is furnished by the glands that secrete it. It only remains long enough in the mouth, to mix with a small quantity of mucus, and absorb a certain portion of atmospheric air, to render it frothy.

"On the other hand, the saliva of an individual, whose mucous system furnishes a large quantity of mucus, is stringy and heavy; is but slightly charged with oxygen, contains a great proportion of azote and sulphur, and stains silver."*

Increased redness and irritability of the mucous membrane of the mouth, is an almost invariable accompaniment of general acidity of these fluids. Excoriation and aphtheous ulcers of it, and bleeding of the gums also frequently result from this condition of the salivary and mucus juices of this cavity.

Anorexia, languor, general depression of spirits, head-ache, diarrhœa, and rapid decay of the teeth, are very common among persons habitually subject to great viscidity of the buccal fluids. It is likewise among subjects of this kind, and particularly when the viscidity is so great as to cause clamminess of these juices, that the green discolouration of the enamel of the teeth, mentioned in a preceding part of this inquiry, is most frequently met with.

### OF THE PHYSICAL CHARACTERISTICS OF THE LIPS.

The indications of the physical characteristics of the lips are more general than local, and the observations of Laforgue and Delabarre on this subject, leave little to be added. I cannot therefore, do much more than repeat what they have said.

"The lips," says, Delabarre, "present marked differences in different constitutions. They are thick, red, rosy or pale, according to the qualities of the arterial blood that circulates through their arteries."

* Vide, Traite de la Seconde Dentition.

Firmness of the lips, and a pale rose-colour of the mucous membrane that covers them, are, according to Laforgue, indicative of pure blood, and as a consequence of a good constitution. Redness of the lips, deeper than that of the pale rose, is mentioned by him, as one of the signs of sanguino-serous blood. Soft, pale lips are indicative of lymphatico-serous dispositions. In these subjects the lips are almost entirely without colour. When there is a sufficiency of blood the lips are firm, though variable in colour, according to the predominancy of the red or serous parts of this fluid.

Hardness of the lips and redness, both of them, and all the soft parts of the mouth are enumerated among the signs of plethora. Softness of the lips, without change of colour in their mucous membrane, is spoken of by this last author as indicative of deficiency of blood; and, softness and redness of the mucous membranes of the lips are signs that the blood is small in quantity and sanguino-serous.

Serous *enemie* and pale blood are indicated by want of colour and softness of the lips, and general paleness of the mucous membrane of the whole mouth.

"The fluids contained in the vessels," says Laforgue, in the three foregoing forms of enema, "yield to the slightest pressure, and leave nothing between the fingers but the skin and cellular tissue."

In remarking upon the signs of the different qualities of the blood, the above mentioned author asserts that the constitution of children, about the age of six years, cannot, by a universal characteristic, be distinguished, but that the lips, as well as other parts of the mouth constantly betoken the "quality of the blood and that of the flesh;" and, "consequently, they proclaim health or disease, or the approach of asthenic and adynamic disorders, which the blood either causes or aggravates."

Again, he tells us that the blood of all children is "superabundantly serous," but that it is redder in those of the second constitution than it is in those of any of the others; and that this is more distinctly indicated by the colour of the lips. "This quality of the blood," says he, "is necessary to dispose all the parts to elongate in their growth. When the proportions of the constituent elements of the blood are just, growth is accomplished without disease. If the proportions are otherwise, then they should be for the preservation of the health, or if one or more of its elements be altered, health no longer exists, growth is arrested altogether, or is performed irregularly. The nutritive matter is imperfect,—assimilation is prevented or impaired. On the other hand, disintegration decomposes the patient; if death does not sooner result, it will consume him by the lesion of some vital organ."*

To the correctness of the foregoing observations every experienced and inquiring dental practitioner can bear witness. The changes produced in the colour of the blood by organic derangements are at once indicated by the colour of the lips.

The accuracy of Laforgue's observations on the indications of the physical characteristics of the lips has been fully confirmed by subsequent writers. Delabarre, in his remarks on the semeiology of the mouth, has added nothing to them.

"The secretion of the lips," says Professor Schill, "has a similar diagnostic and prognostic import to that of the tongue and gums. They become dry in all fevers and in spasmodic paroxysms. A mucous white coating is a sign of irritation or inflammation of the intestinal canal; accordingly, this coating is found in mucous obstructions, in gastric intermittent fever, in mucous fever, and before the gouty paroxysm. A dry brown coating of the lips is a sign of colliquation in consequence of typhus affection; it is accordingly observed in typhus, in putrid fever, in acute exanthems, and inflammations which have become nervous."†

The appearance of the lips, however, do not present so great a variety as those of other parts of the mouth, for the reason that they are not as subject to local diseases, but their general pathognomic indications, are, perhaps, quite as decided.

### OF THE PHYSICAL CHARACTERISTICS OF THE TONGUE.

The appearances of the tongue, both in health and disease, are regarded by physicians as furnishing more correct indications of the state of the constitution and general health, than those of any

*Vide, *Séméiologie Buccale et Buccamancie.*
† Vide, *Pathological Semeiology,* p. 152.

of the other parts of the mouth. It is asserted, however, by others, and those, too, who have the very best opportunities for inspecting the various parts of this cavity, that the lips and gums furnish as plain and decided indications as the tongue. That the state and quality of the blood can be as readily ascertained by an examination of these, as by that of the tongue, is, I believe, undeniable, but that the pathological condition of the body can be, is a question which I shall leave for others to decide.

So far as the quality of the blood and the temperament of the subject are indicated by the colour of the tongue, the remarks that have been made concerning that of the lips will be found applicable here. For, that of the one, is as much influenced by them as is that of the other. It will, therefore, be unnecessary to recapitulate what I have before said.

The effects produced on the mucous membrane of the tongue, by disease in another part, are said to be analagous to those produced on the general integument. So, also, are the changes of its colour, consistence, humidity and temperature similar to those of the skin. We are likewise told that the changes of the coating of it agree with analagous changes of the perspiration, and that these phenomena are more decided in acute than in chronic affections.*

But the diagnostic and prognostic indications of the tongue, vary according to the temperament and constitutional predisposition of the individual. The physician, therefore, should acquaint himself with its appearances in health, to be enabled to determine correctly its indications in disease. He should, likewise, inform himself of the changes that are produced in its appearances by certain morbid conditions of the body. In some subjects it is always slightly furred, especially near its root, and rather dry; in others it is always clean and humid; in some again, it is always red, and in others pale.

Professor Schill divides the signs of the tongue into objective and subjective. To the objective, "the changes of size, form, consistence, colour, temperature, secretion," and those of its "motion belong;" and "to the subjective, the anomalous sensations of taste." I do not know, however, that any advantage can be derived from this classification.

* Vide, Professor Schill's Semeiology.

In enumerating the pathognomic signs of the tongue, this author says that hypertrophy, inflammation or congestion, may occasion its enlargement; and that inflammatory swelling of it, when arising from acute diseases, such as "angina, pulmonary inflammation, measles, plague, or variola, yields an unfavorable prognosis. Even non-inflammatory swelling of the tongue, is a dangerous phenomenon, in acute diseases, especially cerebral, which are combined with coma. If it be the consequence of mercury, of the abuse of spirituous drinks, of gastric inflammation, of chlorosis, of syphilis, or if it occur in histeria or epilepsy, the prognosis is not dangerous; but the disease is always the more tedious where the tongue swells than where it does not. It is enlarged, also, by degenerescence and cancer."

"Diminution of the size of the tongue takes place where there is considerable emaciation. In this case it continues soft and movable. If, in acute states, the tongue becomes small, and is, at the same time, hard, retracted, and pointed; the irritation is very great, and the prognosis bad. This sign occurs more especially in typhus, in the oriental cholera, in inflammation of the lungs, and in acute, cerebral affections. In histeria and epilepsy, this phenomenon has no unfavourable import."

Internal maladies, he says, seldom causes the form of the tongue to change, but, that the slightest change arising from chronic irritations of the stomach, chronic dyspepsia, and acute exanthems, is enlargement of its papillæ. In cases of protracted dyspepsia, the edges of the tongue sometimes crack, and in paralysis and epilepsy, it becomes elongated.

In acute diseases, a soft tongue is a favourable indication, and flaccidity of it, that of debility.

Humidity of the tongue, he tells us, is a favourable sign, and that dryness of it occurs in acute or violent inflammations and irritations, and more particularly when seated in the intestinal canal, and respiratory organs, as in the case of diarrhœa, typhus, pneumonia, gangrene of the lung, pleuritis, peritonitis, enteritis, catarrhus gastricus, gastritis, inflammation of joints, &c. Among the higher degrees of dryness, he enumerates the rough, the fissured and burnt tongue, as furnishing still more unfavourable indications, informing us at the same time that if these be not accompanied by thirst, they prognosticate a fatal termination. The abatement and crisis of the disease is indicated by the tongue's becoming moist.

Dr. Bell, of Philadelphia, in a note to Professor Schill's observations on the tongue, says, "a rough, and dry, and even furred tongue, is seen in some dyspeptic persons, who sleep with the mouth open; and although it indicates an irritation of the digestive organs, it is not of a bad augury." Bilious persons, not unfrequently, though not troubled with any manifest symptoms of gastric or intestinal derangement, or any other apparent functional disturbance, have a furred tongue in the morning.

Paleness of the tongue, we are told by Professor Schill, is a sign of a serous condition of the blood, of chlorosis, of great loss of blood, of chronic disorders, of sinking of the strength in acute maladies, assuming a "nervous form, as typhus and scarlatina maligna. It is also found," says he, "in enteritis and dysentery, when but little fever is present." He infers from this, that paleness of the tongue is caused by the "drawing of the fluids downwards," but it is often observed in persons who enjoy tolerably good health. Lymphatic dispositions, as has been before remarked, are peculiarly subject to it.

Again he observes, that a very red tongue is indicative of "violent inflammation, mostly of the intestinal canal, but also of the lungs and of the pharynx and acute exanthems." He regards the prognosis as bad, when a furred tongue "in acute diseases of the intestinal canal becomes clean and very red," if the change be not accompanied with the return of the patient's strength. "But," he continues, "if the debility is not considerable, and the tongue becomes clean and very red, whilst other febrile symptoms continue, a new inflammation may be expected." But, even in affections like these, the redness of the tongue is always more considerable in sanguinous, than it is in lymphatic or lymphatico-serous subjects. So that in the forming of a prognosis from this sign, the temperament of the individual should never be overlooked.

Proceeding with the description of the signs of this organ, he says, "the tongue becomes a blackish-red and bluish-red in all serious disturbances of the circulation and respiration, as also in severe diseases of the lungs and heart, as catarrhs, suffocations, asthma, extensive inflammation of the lungs, carditis, Asiatic cholera, plague, confluent small-pox, and putrid fevers. It becomes black and livid in cases of vitiation of the blood, more especially in scurvy, at the setting in of gangrene, and in phthisis, when death is near at hand."

Among the diseases mentioned as giving rise to an increase of the temperature of the tongue are glossitis, violent internal inflammation and typhus; and, coldness of this organ, is observed to take place in Asiatic cholera, and at the approach of death.

The signs from the secretion of the tongue are thus enumerated. A clean and moist tongue are favourable indications, but a clean, dry and red tongue, as are seen in slow nervous fevers, acute exanthems and plague, are bad auguries. A furred or coated tongue is said to occur chiefly, in intestinal disorders, diseases of the lungs, skin, and in rheumatic affections. The coating is said to vary in "colour, thickness, adherence, and extent," and different kinds of secretion from the mucous membrane of this organ are mentioned as occurring in different diseases, and it should have been added in the same disease in different temperaments.

After describing the various kinds of coating on the tongue, together with their respective indications, which it is not necessary here to enumerate, the occurrence of false membranes and pustules, resulting from peculiar forms of mucous secretion are next mentioned. The former show themselves either as small white points, or large portions, and sometimes they are said to envelope the whole tongue. Their colour is "sometimes white, sometimes yellow and sometimes red," and the greater the surface covered by them, the more unfavourable the prognosis is regarded. "Pustules on the tongue," says the author, "are sometimes idiopathic, but in most cases symtomatic. They are either distinct or confluent; the confluent are the worse. Those which are hardish and dry, and also those which are blue, and those of a blackish appearance, which sometimes occur in acute diseases, are of an unfavourable import." On the other hand, those which have a whitish, soft, moist and semi-transparent appearance, are less unfavourable, and when the eruption or aphthæ is repeated, it portends a longer continuance of the malady. To the following diseases, they are mentioned as being frequent accompaniments; namely, gastritis, catarrhs, enteritis, metritis, dysentery, cholera infantum, peritonitis, intermittent and typhus fevers, pluritis, pneumonia, and the third stage of pulmonary consumption. Their prognosis is said to be favourable, when "they appear with critical discharges after the seventh day," and unfavourable, when they occur as a consequence of a general sinking of the physical powers of the body.*

But it is unnecessary to enumerate all of the pathognomic indications of the various morbid phenomena that have been described by semeiologists, I have already noticed more of them, than it was my my intention at first to have done. I shall, therefore, conclude the present inquiry, by simply observing, that the indications furnished by the physical characteristics, of not only the tongue, but those also, of the teeth, the gums, salivary calculus, the lips and fluids of the mouth, are, as I have here endeavoured to show, essential to the successful exercise of the duties of both the dental and medical practitioner.

* Vide, Professor Schill's Semeiology.

